# Phase-synchronized brain connectivity during emotion regulation: attachment as a moderator

**DOI:** 10.1093/scan/nsaf069

**Published:** 2025-07-01

**Authors:** Marcos Domic-Siede, Mónica Guzmán-González, Romina Ortiz, Sara Hernández, Catalina Carvallo

**Affiliations:** Escuela de Psicología, Universidad Católica del Norte, Antofagasta, 1270709, Chile; Escuela de Psicología, Universidad Católica del Norte, Antofagasta, 1270709, Chile; Escuela de Psicología, Universidad Católica del Norte, Antofagasta, 1270709, Chile; Escuela de Psicología, Universidad Católica del Norte, Antofagasta, 1270709, Chile; Escuela de Psicología, Universidad Católica del Norte, Antofagasta, 1270709, Chile

**Keywords:** attachment theory, emotion regulation, cognitive reappraisal, expressive suppression, brain connectivity

## Abstract

Emotion regulation is essential for modulating emotional experiences. According to studies in attachment, individual differences in attachment anxiety and avoidance may lead to difficulties in the strategies used to regulate emotions. These dispositions may shape the neural mechanisms underlying regulation. This study examined oscillatory brain connectivity during two strategies—cognitive reappraisal and expressive suppression—across delta (1–3 Hz), theta (4–8 Hz), alpha (9–12 Hz), and beta (15–30 Hz) bands. We also tested whether attachment orientations modulate these connectivity patterns. Sixty adults completed an emotion regulation task while electroencephalographic (EEG) was recorded. Connectivity was estimated using the debiased weighted Phase Lag Index (wPLI). Linear mixed-effects models assessed the effects of condition, attachment, and their interaction on connectivity between frontal or central electrodes and the rest of the scalp. Reappraisal increased theta-band connectivity between frontal and distributed sites, while suppression enhanced beta connectivity involving frontal and central electrodes. Higher attachment anxiety was associated with reduced theta connectivity during reappraisal, and higher avoidance predicted increased beta connectivity during suppression. These findings suggest that emotion regulation strategies engage distinct oscillatory networks, modulated by interpersonal dispositions. Theta connectivity may reflect top-down control processes in reappraisal, while beta connectivity may support inhibition during suppression.

## Introduction

Attachment theory (Bowlby 1969, 1973, Ainsworth 1989) offers a foundational framework for understanding individual differences in emotion regulation (ER), proposing that early caregiver interactions shape internal working models that influence emotional responses across the lifespan (Cassidy 1994; Mikulincer and Shaver 2016, 2019). Attachment is commonly described along anxiety and avoidance dimensions representing tendencies to hyperactivate or deactivate the attachment system, respectively (Brennan et al. 1998): Individuals high in anxiety tend to be hypervigilant and ruminate on negative emotional cues (Mikulincer and Shaver 2007; Cassidy and Shaver 2016), whereas those high in avoidance tend to suppress affect and disengage from emotional stimuli (Shaver and Mikulincer 2002; Vrtička and Vuilleumier 2012). These patterns also modulate neurophysiological processes involved in ER (Domic-­Siede et al. 2024a, 2025a).

One of the most influential theoretical frameworks for understanding ER is James Gross’s process model (1998, 2015), which organizes regulatory strategies according to the temporal unfolding of emotional responses. It distinguishes between antecedent-focused strategies—such as situation selection, attentional deployment, and cognitive reappraisal—and response-focused strategies like expressive suppression. These strategies differ in both timing and goals: some aim to cognitively reframe or engage with emotional situations, while others seek to inhibit or avoid emotional expression (Gross 2001, 2002, 2015). The model has guided empirical research on the neural basis of ER, emphasizing that the effectiveness and cognitive demands of each strategy can vary across individuals and contexts (Gross 2024). Within this framework, the present study focuses on two commonly used strategies—reappraisal and suppression—situated at distinct points in the regulatory process, and examines their neural implementation as a function of individual attachment orientations.

Emotion regulation allows individuals to modulate emotional states in response to internal and external demands (Gross 1998, Gross and John 2003). Reappraisal and suppression involve distinct neurocognitive processes and oscillatory dynamics (Ertl et al. 2013, Zouaoui et al. 2023, Domic-Siede et al. 2024a). Brain oscillations—rhythmic brain activity measurable via electroencephalography (EEG)—reflect communication across brain regions and are categorized into frequency bands (delta, theta, alpha, beta, gamma), supporting cognitive functions such as attention, memory, inhibitory control, and ER (Buzsáki and Draguhn 2004).

### Brain oscillations and emotion regulation

Brain oscillations are central to the neural mechanisms of ER. Cognitive reappraisal, involving reinterpretation of emotional stimuli, is consistently associated with increased frontal theta activity (3–6 Hz), reflecting cognitive control and flexibility (Ertl et al. 2013, Zouaoui et al. 2023, Domic-Siede et al. 2024a). Ertl et al. (2013) observed sustained low frontal theta increases 3–5 s post-regulation onset, while Wei et al. (2017) reported theta decreases during downregulation, illustrating the complexity of these dynamics. Distancing and distraction strategies reduce occipital theta early in regulation, likely due to attentional shifts (Uusberg et al. 2014, Sulpizio et al. 2021). Moreover, training in ER has been linked to resting-state increases in theta and delta, suggesting neural plasticity (Lapomarda et al. 2022).

Expressive suppression, which inhibits emotional expression, has been associated with changes in central beta activity (15–30 Hz), reflecting motor inhibition and response control (Engel and Fries 2010, Heinrichs-Graham and Wilson 2016, Domic-Siede et al. 2024a). Beta rhythms are tied to sensorimotor stability and cognitive maintenance (Gilbertson et al. 2005).

Alpha oscillations (8–13 Hz) remain more complex. Typically linked to attentional gating and cortical inhibition (Sadaghiani and Kleinschmidt 2016), alpha activity responds more to emotional induction than to regulation itself (Zouaoui et al. 2023). Still, alpha connectivity between parietal and frontal regions supports top-down control required to sustain cognitive regulation goals (Choi et al. 2016, Papousek et al. 2017). During emotional induction, occipital alpha decreases due to heightened attention to salient stimuli (De Cesarei and Codispoti 2011, Dmochowski et al. 2012), whereas alpha may shift dynamically during reappraisal to meet regulatory demands. Thus, oscillatory patterns offer valuable insight into the attentional and cognitive mechanisms underpinning ER.

### Brain connectivity in emotion regulation

While most ER research has focused on ERP amplitudes (Hajcak and Nieuwenhuis 2006, Moser et al. 2006, Soto et al. 2011, Murata et al. 2013, Yuan et al. 2015, MacNamara et al. 2022, Yan et al. 2022) and power modulations (Ertl et al. 2013, Zouaoui et al. 2023, Domic-Siede et al. 2024a), brain connectivity—temporal coordination of neural oscillations—offers complementary insights into network-level communication during regulation. Frontal–posterior theta phase connectivity supports sensory integration and executive control (Frank and Cavanagh 2014), suggesting that reappraisal may rely on enhanced theta synchrony between frontal and parietal sites.

In suppression, both beta power reductions (possibly reflecting disengagement from automatic motor tendencies) and connectivity increases have been reported, reflecting inhibition of motor responses (Gilbertson et al. 2005, Swann et al. 2009). Connectivity analyses thus capture frequency-specific interactions underlying regulatory strategies. Theta synchrony facilitates selective routeing of information (Womelsdorf et al. 2010) and temporal coordination of neuronal firing (Benchenane et al. 2010), promoting goal-directed behaviour.

Multimodal EEG-fMRI studies show that reappraisal engages a distributed network—including lateral orbitofrontal, opercular, and superior parietal regions—emphasizing the need for broad neural coordination (Li et al. 2022). Additionally, frontal theta has been linked to communication with the cingulate cortex, functioning as a monitoring hub (Shackman et al. 2011; Cohen and Cavanagh 2011).

### Attachment theory and its neural correlates

Recent research has explored how attachment orientations shape neural processes in ER. Individuals high in attachment anxiety often display increased limbic reactivity and reduced recruitment of frontal control regions (Vrtička and Vuilleumier 2012, Long et al. 2020), potentially reflected in diminished frontal theta power and connectivity during reappraisal (Ertl et al. 2013, Domic-Siede et al. 2024a). In contrast, avoidantly attached individuals tend to rely on suppression and may engage brain areas related to inhibitory control (Vrtička and Vuilleumier 2012; Long et al. 2020). Although direct evidence linking beta connectivity to avoidance is limited, findings on motor inhibition suggest a role for beta oscillations in supporting suppression (Gilbertson et al. 2005; Swann et al. 2009).

Future studies should examine how oscillatory patterns interact with attachment dimensions to uncover neural pathways underlying stable ER tendencies. As emphasized by MacNamara et al. (2022), accounting for individual differences such as attachment is crucial for advancing the understanding of ER mechanisms, both conceptually and methodologically. Connectivity analyses can further elucidate frequency-specific interactions supporting these differences.

### The present study

This study investigates brain connectivity in theta, alpha, and beta bands during cognitive reappraisal and expressive suppression. It also examines whether attachment anxiety and avoidance moderate these connectivity patterns.

Based on prior literature, we hypothesize that (i) Reappraisal will increase theta connectivity, particularly between frontal and other electrode sites, reflecting cognitive control processes; (ii) Suppression will increase beta connectivity, especially from central electrodes, due to motor-inhibitory demands; and (iii) Alpha connectivity may increase during regulation, reflecting attentional or control engagement, though this remains exploratory given the mixed evidence on alpha dynamics in regulation contexts.

Regarding attachment orientations, we hypothesize that (iv) Higher attachment anxiety predicts reduced theta connectivity during reappraisal, reflecting impaired top-down control; and (v) Higher attachment avoidance predicts increased beta connectivity during suppression, consistent with reliance on deactivating strategies.

By integrating connectivity analyses with attachment theory, this study aims to elucidate how interpersonal dispositions shape the neural implementation of ER.

## Materials and methods

### Participants

A purposive, non-probabilistic sampling strategy was used to recruit adults over 18 years old, right-handed, with normal or corrected-to-normal vision. All participants gave written informed consent voluntarily. Recruitment was carried out through digital platforms of the School of Psychology at Universidad Católica del Norte, Chile.

Of the initial 65 participants, five were excluded due to noisy EEG or technical issues, yielding a final sample of 60 (59 Chilean, 1 Colombian): 30 female (20–52 years, M = 27.53, SD = 9.31), 27 male (18–58 years, M = 29.59, SD = 10.71), and three undisclosed (19–22 years, M = 21.00, SD = 1.73) (Table 1). A priori power analysis using G*Power 3.1.9.2 (Faul et al. 2007) for repeated-measures ANOVA (three within-subject conditions, one between-subject factor), with *α* = .05, power = .85, and *f* = 0.20—based on EEG studies on emotional modulation (e.g. Uusberg et al. 2014)—indicated a minimum of 48 participants. The final sample exceeded this threshold.

**Table 1.** Sociodemographic characteristics of the participants.

**Variables** total participants (*n*) = 60	*n*	%	Mean (SD)
**Age**			28.13 (9.85)
**Sex**			
Female	27	45.00	
Male	30	50.00	
Other/Prefer not to say	3	5.00	
**Education**			
Completed High School	5	8.33	
Completed Technical Education	6	10.00	
Completed Higher Education	8	13.33	
Incomplete University/Technical Education	35	58.33	
Postgraduate Studies (Master’s, Doctorate, or equivalent)	6	10.00	
**Socioeconomic status**			
Low	7	11.67	
Lower-middle	15	25.00	
Upper-middle	29	48.33	
High	9	15.00	
**Nationality**			
Chilean	59	98.33	
Colombian	1	1.67	

SD = Standard Deviation.

To assess sensitivity, a post-hoc power analysis considered two significant interactions from our linear mixed-effects models: attachment anxiety × reappraisal in the theta band (*β* ≈ –0.025 to –0.029), and attachment avoidance × suppression in the beta band (*β*  ≈  0.023 to 0.025). Given *N* = 60, *α* = .05, and a repeated-measures ANOVA framework with three conditions, the design was sensitive to effects of *f* ≈ 0.18. Crucially, these effects remained significant after FDR correction, supporting statistical robustness.

Ethical approval was granted by the Scientific Ethics Committee of Universidad Católica del Norte (Resolutions No. 099/2021 and 037/2023). All procedures adhered to the Declaration of Helsinki.

### Instruments

#### Experiences in close relationships questionnaire (ECR-12)

Attachment was assessed using the Spanish 12-item version of the ECR-12, validated for Chile by Guzmán-González et al. (2020). This self-report instrument measures attachment anxiety and avoidance through two six-item subscales (e.g. ‘I feel uncomfortable opening up to my partner’ for avoidance; ‘If I cannot get my partner to show interest in me, I get upset or angry’ for anxiety) (Brennan et al. 1998). Items are rated on a 7-point Likert scale (1 = strongly disagree, 7 = strongly agree). Higher scores indicate greater attachment insecurity (Guzmán-González et al. 2023). The scale showed good internal consistency in this sample (*α* = .80 for anxiety, *α* = .77 for avoidance), consistent with psychometric evidence from both the 12- and 36-item Chilean versions (Spencer et al. 2013).

#### EEG data acquisition

Brain activity was recorded using a scalp EEG Biosemi^Ⓡ^ System (www.biosemi.com) consisting of 30 channels positioned on the scalp and two on the mastoids (international 10/20 system) (Keil et al. 2014). Data were sampled online at 2.048 Hz and referenced to CMS/DRL active electrodes. Electrode impedance was kept below 20 kΩ.

### Emotion regulation task

To assess ER, we employed an experimental task adapted from Ochsner et al. (2004), Vrtička et al. (2012), and recent work by Domic-Siede et al. (2023a, 2024a, 2024b), implemented in Presentation^®^ (Version 18.0, Neurobehavioral Systems). The task included 60 IAPS images (Lang et al. 2005): 45 negative and 15 neutral, selected based on prior ER studies (Moser et al. 2009, Schlumpf et al. 2019, Domic-Siede et al. 2023a) (Supplementary Tables S1–S3).

Participants completed three conditions: *Natural*, *Reappraise*, and *Suppress* (Domic-Siede et al. 2023a), with stimuli presented on a 23.6" ASUS VG248QE monitor. A training phase with three blocks of three practice trials per condition preceded the task, including visual aids and instruction on arousal rating using the Self-Assessment Manikin (SAM; Bradley and Lang 1994). In the *Natural* condition, participants observed images and allowed emotions to arise naturally. In *Reappraise*, they used cognitive reappraisal to reinterpret the image (e.g. imagining a positive or fictional outcome; Ochsner et al. 2004). In *Suppress*, they inhibited facial and bodily expressions.

**Figure 1.**
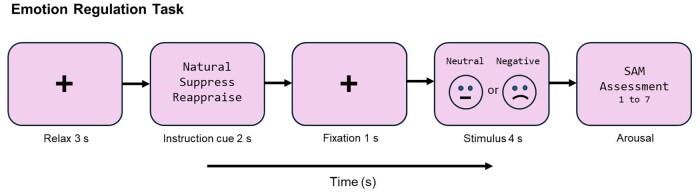
Emotion regulation paradigm. The figure illustrates the temporal structure of a representative trial in an experimental paradigm designed to assess the impact of emotion regulation strategies on affective responses. Each trial begins with a 3-s relaxation phase, marked by a fixation cross. This is followed by a 2-s instruction cue that directs participants to engage in one of three strategies: respond naturally, suppress their emotional reaction, or reappraise the emotional meaning of the forthcoming stimulus. A 1-s fixation period then precedes the stimulus presentation, during which a neutral or negative image is displayed for 4 s. Immediately afterward, participants rate their level of experienced arousal on a 7-point Likert scale, where one represents low emotional intensity and seven represents high intensity.

After each image, participants rated emotional intensity on a 7-point SAM scale (1 = low, 7 = high), responding to the prompt: *‘Indicate the intensity of your emotional response to the image you just saw.’* The task comprised 12 randomized blocks of five trials each. *Natural* included 30 trials (15 neutral, 15 negative: *Nat-neutral* and *Nat-negative*), while *Reappraise* and *Suppress* included 15 negative-image trials each. All participants viewed the same fixed set of images per condition—i.e. each image was uniquely assigned to *Natural*, *Reappraise*, or *Suppress*. The order of the 12 blocks and the sequence of images within each block were randomized for each participant. This design ensured randomization both at the block level and within blocks on a trial-by-trial basis. Short breaks were included between blocks to minimize fatigue. Each trial followed a fixed sequence (Fig. 1): (i) 3-s fixation cross; (ii) 2-s instruction screen; (iii) 1-s fixation; (iv) 4-s image presentation; and (v) arousal rating screen. This sequence repeated across all blocks.

### Procedure

An experimental design was used with participants recruited via snowball sampling. The ECR-12 was completed digitally either before or after the ER task in a counterbalanced order to control for sequence effects. During the task, participants viewed IAPS images and rated their arousal using the SAM scale after each trial. The full session lasted approximately 25 minutes.

### Data analysis

#### Behavioural analysis

Behavioural data were analysed using GraphPad Prism 8 and MATLAB R2022b. Descriptive statistics summarized participants’ self-reported arousal ratings. The Shapiro–Wilk test assessed normality (Mishra et al. 2019), revealing significant deviations and skewness; thus, non-parametric methods were applied.

A Friedman test was used to compare arousal ratings across ER conditions, as a non-parametric alternative to repeated-measures ANOVA (Field 2013). When significant effects emerged, Dunn’s post-hoc tests were conducted, and Cohen’s d was calculated to estimate the magnitude of pairwise differences.

#### EEG signal preprocessing

EEG preprocessing followed established protocols (Domic-Siede et al. 2021, 2023b, 2024a). A high-pass FIR filter at 1 Hz was applied to remove slow drifts, followed by a low-pass filter at 40 Hz to eliminate high-frequency noise. Data were then downsampled to 500 Hz.

Epochs were segmented from −1000 ms to 4000 ms relative to IAPS image onset across four conditions: Natural Neutral, Natural Negative, Reappraise, and Suppress. Trials with gross artefacts were visually rejected. Average retained trials were high and comparable: Neutral (M = 14.45, SD = 1.10), Negative (M = 14.42, SD = 1.03), Reappraise (M = 14.33, SD = 1.22), Suppress (M = 14.58, SD = 0.74), ranging from 8 to 15 per condition. A Friedman test indicated no significant differences in trial retention across conditions, *χ*^2^(3) = 2.36, *P* = .501; Dunn’s post-hoc tests confirmed all pairwise comparisons were non-significant (adjusted *P* values > .9999), suggesting balanced trial availability.

Artifact correction was refined using Independent Component Analysis (ICA; Logistic Infomax algorithm, Bell and Sejnowski 1994). Components associated with non-neural activity (e.g. eye blinks, muscle noise, cardiac signals) were identified using ICLabel (Pion-Tonachini et al. 2019) and visually inspected. Between five and eight components were removed per participant (M = 6.47, SD = 1.10), not exceeding ∼25% to preserve neural signal integrity (Jung et al. 2000). This pipeline optimized signal-to-noise ratio while maintaining spatial fidelity for subsequent analyses.

#### Brain EEG connectivity analysis

Connectivity analyses were conducted using custom MATLAB R2022b scripts, with EEG data processed in EEGLAB (Delorme and Makeig 2004) and FieldTrip (Oostenveld et al. 2011). Analyses focused on the 300–4000-ms post-stimulus window, aligned with the onset of the Late Positive Potential (LPP)—a marker of sustained emotional processing (e.g. Hajcak and Nieuwenhuis 2006, Moser et al. 2006, Soto et al. 2011, Murata et al. 2013, MacNamara et al. 2022, Ramos-Henderson et al. 2024, Domic-Siede et al. 2025a)—and supported by prior evidence of theta increases during reappraisal and beta decreases during suppression (Ertl et al. 2013, Domic-Siede et al. 2024a). Accordingly, we defined the 300–4000-ms interval to encompass the core phase of ER, excluding early perceptual components and enhancing sensitivity to oscillatory signatures associated with distinct regulatory strategies.

EEG epochs per condition (Reappraise, Suppress, Neutral, Negative) were segmented (300–4000 ms), and spectral analysis was performed using multitaper FFT (mtmfft with DPSS, ±2 Hz smoothing) to extract delta (1–3 Hz), theta (4–8 Hz), alpha (9–12 Hz), and beta (15–30 Hz) bands. Phase synchrony was computed using debiased weighted Phase Lag Index (wPLI-debiased; Vinck et al. 2011), which minimizes volume conduction effects.

Due to the 32-channel setup, connectivity analyses were conducted at the scalp level, as source reconstruction reliability is limited in low-density configurations (Grech et al. 2008, Michel and Brunet 2019). Although wPLI mitigates volume conduction, scalp-level estimates still lack anatomical precision (Lai et al. 2018), but provide robust assessments of phase-based connectivity.

For each participant and condition, a square matrix of average wPLI-debiased values was computed between electrode pairs. Group-level connectivity matrices were derived by averaging individual matrices. To identify connectivity increases, matrices from Reappraise, Suppress, and Negative conditions were contrasted against Neutral. A data-driven threshold (95th percentile of all positive differences) was applied per frequency band to retain only meaningful increases; negative values were excluded, and subthreshold values attenuated.

Individual connectivity values were then computed by averaging each participant’s wPLI-debiased values across electrode pairs that surpassed the group-level threshold for a given frequency band. This approach anchored individual metrics in robust, group-informed patterns, enhancing sensitivity while minimizing spurious influences.

Connectivity was examined within two regions of interest (ROIs): **frontal** (Fp1, Fp2, Fpz, Fz, F3, F4, F7, F8, AF3, AF4, AF7, AF8, FC1, FC2, FC5, FC6) and **central** (Cz, C3, C4, CP1, CP2, CP5, CP6) (Supplementary Table S4). For each participant and condition, mean connectivity within each ROI was calculated by averaging the values of all masked electrode pairs containing at least one electrode in the respective ROI. These ROI-specific metrics were analysed via Linear Mixed-Effects Models to assess how attachment anxiety and avoidance modulated neural connectivity during ER.

#### Linear Mixed-Effects model for brain connectivity analysis

To examine how attachment anxiety and avoidance modulate functional brain connectivity during ER, we applied linear mixed-effects models (LMMs) to debiased wPLI values from frontal and central ROIs across delta, theta, alpha, and beta bands. Linear mixed-effects models were chosen for their capacity to handle hierarchical, repeated-measures data while preserving statistical power (Baayen et al. 2008, Barr et al. 2013, Matuschek et al. 2017).

Separate models were estimated for each ROI–frequency band combination. Each included fixed effects for Condition (Negative, Reappraise, and Suppress), one attachment dimension (Anxiety or Avoidance), and their interaction, plus a random intercept per participant to account for individual baseline variability.

Model structure:


$$\mathrm{wPLI}\;\mathrm{Connectivity}y_{\mathrm{ij}}=\beta 0+\beta 1({\mathrm{Condition}}_{\mathrm{ij}})+\beta 2(\mathrm{Attachme}{\mathrm{nt}}_{\mathrm{ij}})+\beta 3({\mathrm{Condition}*\mathrm{Attachment}}_{\mathrm{ij}})+b_{0i}+\epsilon_{\mathrm{ij}}$$

Where *i* indexes participants, *j* indexes condition-level observations, *βs* represent fixed effects, $b_{0i}$ is the random intercept for subject *i*, and $\epsilon_{\mathrm{ij}}$ is the residual error.

Model fit was evaluated using AIC, BIC, and likelihood ratio tests. Full results are available in Supplementary Tables S5–S6. This approach allowed us to assess whether ER-related changes in connectivity are moderated by attachment orientations.

### Data accessibility

All MATLAB scripts used for EEG connectivity and LME analyses are openly available at: https://github.com/domic-siede/EEG_Connectivity_ER_Study/. The data underlying this article will be shared upon reasonable request to the corresponding author.

## Results

### Self-Reported arousal across emotion regulation conditions

Self-reported arousal ratings (1–7 scale) were compared across four conditions: Natural Neutral, Reappraise, Suppress, and Natural Negative. Descriptive data showed a progressive increase from Natural Neutral (M = 2.09, SD = 0.98) to Natural Negative (M = 2.91, SD = 1.38), with intermediate values for Reappraise (M = 2.43, SD = 1.23) and Suppress (M = 2.60, SD = 1.31) (Supplementary Table S7).

Due to non-normal distributions (Shapiro–Wilk; Supplementary Table S8), a Friedman test was used and revealed a significant effect of Condition, *χ*^2^(3) = 50.45, *P* < .0001. Post-hoc Dunn’s tests showed higher arousal in Suppress (*P* = .0036, *d *= 0.44) and Natural Negative (*P* < .0001, *d *= 0.68) compared to Natural Neutral. Reappraise did not differ from Natural Neutral (*P* = .4626, *d *= 0.30) or Suppress (*P* = .5794, *d *= 0.13), but Natural Negative elicited higher arousal than both Reappraise (*P* < .0001, *d *= 0.37) and Suppress (*P* = .0069, *d *= 0.23) (Supplementary Table S9).

These findings indicate that Suppress and Natural Negative conditions increased arousal relative to a neutral baseline, while Reappraise effectively reduced arousal compared to Natural Negative, supporting its regulatory efficacy in this paradigm.

### Brain connectivity across frequency bands

We examined condition-related changes in functional connectivity using debiased wPLI across delta (1–3 Hz), theta (4–8 Hz), alpha (9–12 Hz), and beta (15–30 Hz) bands. Group-level difference matrices were computed relative to the Neutral condition and thresholded using the 95th percentile of all positive ΔwPLI values across participants and conditions.

In the delta band, Suppress showed the highest number of suprathreshold pairs (28), while Reappraise and Negative showed fewer (four and five, respectively; Supplementary Table S10 and Fig. S1).

**Figure 2.**
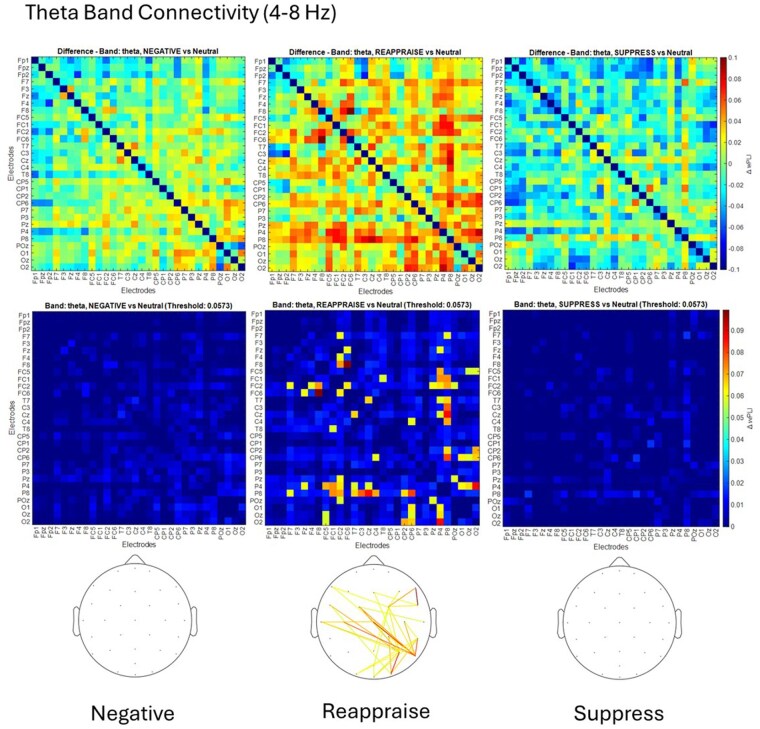
Theta band connectivity (4–8 Hz) during emotion regulation conditions. *Top row*: Group-level difference matrices showing changes in debiased weighted Phase Lag Index (wPLI) connectivity in the theta band for each emotion regulation condition (Negative, Reappraise, and Suppress) relative to the Neutral baseline. Warmer colours indicate stronger increases in phase synchrony between electrode pairs (ΔwPLI). Colorbar range: –0.1 to 0.1. *Middle row*: Thresholded difference matrices based on the 95th percentile of all positive differences across participants and conditions. Only electrode pairs with ΔwPLI exceeding the data-driven threshold (0.0573) are shown, with values below this cut-off attenuated or masked. Colorbar range: 0 to 0.09. *Bottom row*: Scalp maps depicting the topographical distribution of suprathreshold functional connections in each condition. Coloured lines represent electrode pairs showing increased theta-band connectivity relative to Neutral, with warmer hues reflecting higher ΔwPLI values. These results indicate that cognitive reappraisal elicited the most prominent theta-phase synchronization, whereas suppression and passive viewing conditions showed minimal changes in connectivity.

In the theta band, Reappraise exhibited the most widespread connectivity (35 suprathreshold pairs), with no suprathreshold pairs observed for Suppress or Negative (Fig. 2; Supplementary Table S11).

In the alpha band, Suppress again had the most suprathreshold connections (29), followed by Reappraise (12) and Negative (4) (Supplementary Figure S2 and Table S12).

**Figure 3.**
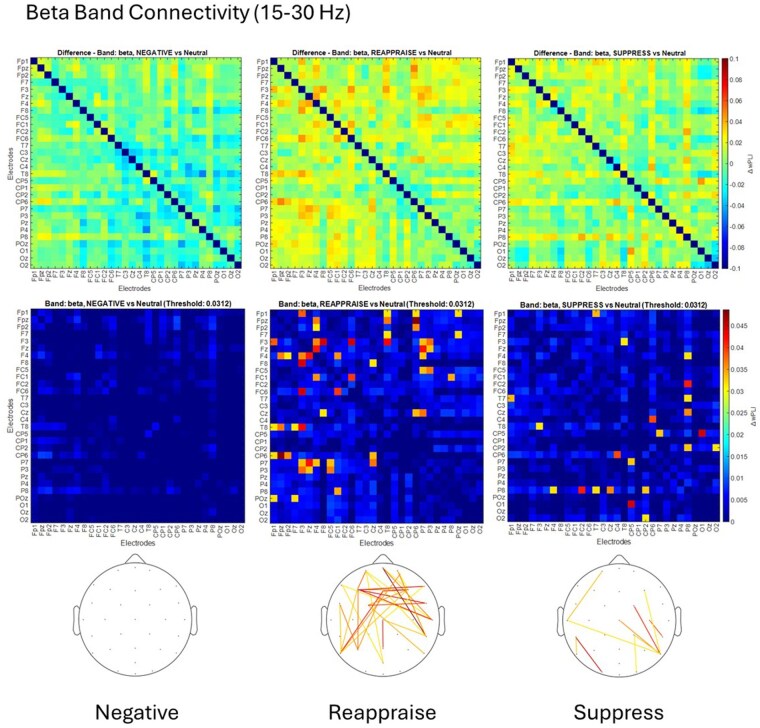
Brain connectivity in the beta band (15–30 Hz) during emotion regulation. *Top row*: Group-level difference matrices showing changes in debiased weighted Phase Lag Index (wPLI) connectivity in the beta band for each emotion regulation condition (Negative, Reappraise, Suppress) relative to the Neutral baseline. Warmer colours indicate stronger increases in phase synchrony between electrode pairs (ΔwPLI). Colorbar range: –0.1 to 0.1. *Middle row*: Thresholded difference matrices based on the 95th percentile of all positive differences across participants and conditions. Only electrode pairs with ΔwPLI exceeding the data-driven threshold (0.0312) are shown, with values below this cut-off attenuated or masked. Colorbar range: 0 to 0.045. *Bottom row*: Scalp maps depicting the topographical distribution of suprathreshold functional connections in each condition. coloured lines represent electrode pairs showing increased beta-band connectivity relative to Neutral, with warmer hues reflecting higher ΔwPLI values. These results reveal widespread beta-phase synchronization during cognitive reappraisal, moderate increases during suppression, and minimal changes under passive viewing of negative stimuli.

In the beta band, Reappraise showed extensive connectivity (29 pairs), Suppress had moderate connectivity (10 pairs), and Negative showed none (Fig. 3; Supplementary Table S13).

Topographical distributions of suprathreshold connections are shown in each figure’s bottom row. Full ΔwPLI matrices and electrode pair values per condition and frequency band are detailed in Supplementary Tables S10–S13.

### Linear mixed-effects models of functional connectivity

We applied LMMs to test the effects of ER condition, attachment anxiety or avoidance, and their interaction on ΔwPLI values across four frequency bands (delta, theta, alpha, beta) and two ROIs (frontal, central). Separate models were run for each band–ROI–attachment combination. Model fit was evaluated using AIC, BIC, log-likelihood, and deviance (Supplementary Tables S5–S6).

#### Models with attachment anxiety as predictor

Model fit criteria for connectivity models including attachment anxiety are shown in Supplementary Table S5. Fixed effects for the frontal ROI are reported in Supplementary Table S14, and for the central ROI in Supplementary Table S15.

- In the **frontal ROI**, significant effects were found in the theta band: Condition: Reappraise (*β* = 0.1393, *P* < .0001, FDR = .0017), Attachment Anxiety (*β* = –0.0247, *P* = .0004, FDR = .0072), and their interaction (β = –0.0291, *P* = .0002, FDR = .0047).
- In the **central ROI**, the theta band again showed significant fixed effects: Condition: Reappraise (*β* = 0.1187, *P* < .0001, FDR = .0011), Attachment Anxiety (*β* = –0.0238, *P* < .0001, FDR = .0011), and their interaction (β = –0.0257, *P* = .0003, FDR = .0043).
- No other frequency bands yielded significant results after FDR correction.

#### Models with attachment avoidance as predictor

Model fit criteria for connectivity models including attachment avoidance are reported in Supplementary Table S6. Fixed effects for the frontal ROI are shown in Supplementary Table S16, and for the central ROI in Supplementary Table S17.

- In the **frontal ROI**, significant effects were observed in the beta band for Condition: Suppress (*β* = –0.0594, *P* < .0001, FDR = .0069) and the Condition × Attachment Avoidance interaction (*β* = 0.0253, *P* < .0001, FDR = .0001). Although the main effect of Attachment Avoidance was statistically significant at the uncorrected level (*β* = 0.0130, *P* = .0103), it did not survive FDR correction (FDR = .2306).
- In the **central ROI**, the beta band also showed significant effects for Condition: Suppress (*β* = –0.0474, *P* = .0015, FDR = .0413) and the Condition × Attachment Avoidance interaction (*β* = 0.0232, *P* < .0001, FDR = .0010). As in the frontal ROI, the main effect of Attachment Avoidance was significant before correction (*β* = 0.0137, *P* = .0051), but not after FDR adjustment (FDR = .1315). Other bands showed no significant effects.

A summary of all significant fixed effects and interactions is presented in Table 2. Full model outputs, including confidence intervals and FDR-corrected *p*-values, are available in Supplementary Tables S14–S17.

**Table 2.** Summary of significant effects in LMM connectivity models (ΔwPLI).

Predictor	ROI	Band	Effect type	Estimate (β)	*P*-value	FDR
Attachment anxiety	Frontal	Theta	Condition: Reappraise	0.1393	<.0001	0.0017
			Attachment Anxiety	–0.0247	.0004	0.0072
			Reappraise × Anxiety	–0.0291	.0002	0.0047
Attachment anxiety	Central	Theta	Condition: Reappraise	0.1187	<.0001	0.0011
			Attachment Anxiety	–0.0238	<.0001	0.0011
			Reappraise × Anxiety	–0.0257	.0003	0.0043
Attachment avoidance	Frontal	Beta	Condition: Suppress	–0.0594	<.0001	0.0069
			Attachment Avoidance	0.0130	.0103	0.2306
			Suppress × Avoidance	0.0253	<.0001	0.0001
Attachment avoidance	Central	Beta	Condition: Suppress	–0.0474	.0015	0.0413
			Attachment Avoidance	0.0137	.0051	0.1315
			Suppress × Avoidance	0.0232	<.0001	0.0010

Estimates (β) represent unstandardized fixed-effect coefficients from linear mixed-effects models predicting changes in debiased weighted Phase Lag Index (ΔwPLI) connectivity relative to the Neutral condition. FDR values correspond to *P*-values corrected for multiple comparisons using the Benjamini-Hochberg procedure. ROI = Region of Interest; Theta = 4–8 Hz; Beta = 15–30 Hz. Only statistically significant effects or interactions are shown (*P* < .05, FDR-corrected).

## Discussion

This study examined oscillatory brain connectivity during ER and the moderating role of attachment anxiety and avoidance. Focusing on delta, theta, alpha, and beta bands, we analysed connectivity during cognitive reappraisal and expressive suppression. Results partially supported our hypotheses: we observed frequency-specific increases—theta connectivity during reappraisal and beta during suppression—and found that attachment dimensions modulated these patterns, highlighting the influence of individual differences on the neural implementation of regulation strategies.

### Theta phase connectivity and reappraisal

Consistent with prior research, reappraisal was linked to widespread increases in theta-band connectivity, especially between frontal and distant electrode sites, supporting its role in cognitive control and neural reorganization (Womelsdorf et al. 2010, Ertl et al. 2013, Cavanagh and Frank 2014, Zouaoui et al. 2023, Domic-Siede et al. 2024a).

Importantly, LMM results showed that higher attachment anxiety was associated with reduced theta connectivity during reappraisal in both frontal and central ROIs, consistent with theories proposing that individuals high in attachment anxiety experience difficulties engaging top-down regulatory control due to hyperactivation of the attachment system (Mikulincer and Shaver 2007, Vrtička and Vuilleumier 2012). This Condition × Anxiety interaction suggests a neural correlate of the regulatory difficulties seen in anxiously attached individuals (Long et al. 2020). Supporting evidence includes elevated LPP amplitudes (Ramos-Henderson et al. 2024, Domic-Siede et al. 2025a) and greater pupil dilation (Domic-Siede et al. 2025b, in press) during reappraisal among those high in attachment anxiety reflecting heightened effort and vigilance.

### Beta phase connectivity and suppression

As hypothesized, suppression was associated with increased beta-band connectivity, especially between central and other scalp regions, consistent with the role of beta oscillations in motor inhibition and sensorimotor stability (Gilbertson et al. 2005, Engel and Fries 2010). These findings align with prior evidence linking suppression to neural systems of inhibitory control (Domic-Siede et al. 2024a).

Furthermore, attachment avoidance significantly moderated this effect: individuals higher in avoidance showed greater beta connectivity during suppression, particularly in frontal and central ROIs. Although the main effect of avoidance did not survive FDR correction, the Condition × Avoidance interaction remained significant, suggesting that avoidant individuals engage inhibitory networks more strongly when regulating emotion. This is consistent with attachment theory, which describes suppression as a typical deactivating strategy among avoidantly attached individuals (Shaver and Mikulincer 2002, Vrtička and Vuilleumier 2012, Mikulincer and Shaver 2019).

### Alpha phase connectivity

Alpha connectivity showed modest increases during reappraisal and suppression, particularly in the Suppress condition, but these did not reach significance in LMMs. This aligns with prior research suggesting that alpha oscillations respond more to emotional induction than regulation (De Cesarei and Codispoti 2011, Zouaoui et al. 2023). Nonetheless, alpha synchrony has been associated with attentional gating and top-down control (Choi et al. 2016, Sadaghiani and Kleinschmidt 2016), potentially reflecting the inhibition of irrelevant neural activity to support regulatory goals. Although not robust, the observed patterns may indicate a general, non-specific role of alpha connectivity in regulatory engagement.

### Theoretical implications

These findings contribute to a growing literature linking oscillatory brain connectivity to ER and interpersonal dispositions. By integrating EEG-based connectivity analyses with attachment theory, this study highlights that the neural implementation of regulatory strategies is not uniform across individuals. Rather, trait-level differences—particularly in how individuals engage in close relationships—can shape the way the brain supports ER.

The observed moderating effect of attachment anxiety on theta connectivity during reappraisal suggests a possible neurobiological pathway underlying the well-documented regulation difficulties in anxious individuals. In contrast, enhanced beta synchrony during suppression among avoidant individuals may reflect their greater capacity to engage inhibitory mechanisms when distancing themselves from emotional engagement.

Building on Gross’s process model of ER (1998, 2015, 2024), our results reveal distinct oscillatory patterns linked to antecedent-focused (reappraisal) and response-focused (suppression) strategies. Reappraisal, which alters the meaning of emotional stimuli, was associated with increased theta connectivity—reflecting cognitive control demands—whereas suppression, which inhibits emotional expression, showed increased beta connectivity—indicative of motor-inhibitory engagement. The modulation of these effects by attachment orientations supports the model’s emphasis on individual and contextual factors. Future research should explore additional regulatory stages, such as attentional deployment and post-regulation outcomes.

### Limitations and future directions

This study has several limitations. First, the modest sample size may reduce statistical power and limit generalizability. Second, connectivity analyses were conducted at the channel level, focusing on frontal and central ROIs. Although this approach captures large-scale coordination, it does not allow for precise source localization. Scalp-level EEG is limited in spatial specificity compared to source-reconstructed networks (Lai et al. 2018), and while wPLI reduces volume conduction effects, spatial inferences remain coarse. Future research should incorporate high-density EEG and source localization methods to improve anatomical accuracy and integrate anatomically informed ROIs.

Third, ERP studies indicate that ER unfolds across multiple stages—anticipation, perception, and evaluation—suggesting future work should explore temporally distinct regulatory phases (Del Popolo Cristaldi et al. 2022). Fourth, the laboratory setting enhances internal validity but limits ecological validity. Emotion regulation in real life is dynamic and context-dependent. Ecological momentary assessment, ambulatory EEG, or immersive paradigms could offer a more realistic understanding (Koval and Kalokerinos 2024, Domic-Siede et al. 2024b).

Fifth, although increases in alpha connectivity were observed, these did not yield significant fixed effects. Given alpha’s role in attentional gating (Sadaghiani and Kleinschmidt 2016), tasks with varying attentional demands may clarify its function in regulatory processes. Sixth, the sample consisted of non-clinical adults, limiting generalizability to clinical populations. As ER is a transdiagnostic process (Aldao et al. 2010, Cludius et al. 2020), future studies should assess whether similar oscillatory patterns and attachment effects emerge in individuals with mood, anxiety, or personality disorders.

Finally, future research should examine how neuromodulatory systems, particularly dopamine and serotonin, contribute to individual differences in ER. Dopamine is linked to reward-based regulation, while serotonin is associated with behavioural inhibition and affective control (Carver et al. 2008, Dayan and Huys 2009, Pizzagalli 2014). Dopaminergic modulation follows an inverted-U function, where both low and high levels impair cognitive control (Cools and D'Esposito 2011), potentially explaining ineffective regulation in some individuals. EEG combined with pharmacological or genetic approaches could clarify how neuromodulators shape neural architecture of ER across varying attachment profiles and clinical conditions.

## Conclusion

This study offers new insights into the neural mechanisms of ER by analysing oscillatory brain connectivity across frequency bands while accounting for attachment orientations. Our findings highlight that:

- **Theta-band connectivity** increased during cognitive reappraisal, particularly in frontal–distributed networks, reflecting cognitive control processes; however, this effect was reduced in individuals with high attachment anxiety, suggesting difficulties in engaging regulatory control networks.
- **Beta-band connectivity** rose during expressive suppression, especially among avoidantly attached individuals, likely indicating engagement of motor-inhibitory and control-related systems in support of deactivating regulatory strategies.

Taken authors declare that the research of attachment-related traits in shaping regulatory brain dynamics and support the utility of oscillatory connectivity as a framework for understanding individual differences in ER. Future research should examine real-time regulation success, cross-cultural variations, and interventions targeting attachment-related neural patterns.

## Supplementary Material

nsaf069_Supplementary_Data

## Data Availability

The data underlying this article will be shared upon reasonable request to the corresponding author.

## References

[nsaf069-B1] Ainsworth MS. Attachments beyond infancy. Am Psychol 1989;44:709–16. 10.1037/0003-066X.44.4.709 2729745

[nsaf069-B2] Aldao A , Nolen-Hoeksema S, Schweizer S. Emotion-regulation strategies across psychopathology: a meta-analytic review. Clin Psychol Rev 2010;30:217–37. 10.1016/j.cpr.2009.11.004 20015584

[nsaf069-B3] Baayen RH , Davidson DJ, Bates DM. Mixed-effects modeling with crossed random effects for subjects and items. J Mem Lang 2008;59:390–412. 10.1016/j.jml.2007.12.005

[nsaf069-B4] Barr DJ , Levy R, Scheepers C et al Random effects structure for confirmatory hypothesis testing: keep it maximal. J Mem Lang 2013;68:255–78. 10.1016/j.jml.2012.11.001 PMC3881361 24403724

[nsaf069-B5] Bell A , Sejnowski TJ. A non-linear information maximization algorithm that performs blind separation. In: Tesauro G, Touretzky D, Leen T (eds), Advances in Neural Information Processing Systems. Cambridge, MA: The MIT Press, 1994, 468–74.

[nsaf069-B6] Benchenane K , Peyrache A, Khamassi M et al Coherent theta oscillations and reorganization of spike timing in the hippocampal-prefrontal network upon learning. Neuron 2010;66:921–36. 10.1016/j.neuron.2010.05.013 20620877

[nsaf069-B7] Bowlby J. Attachment and loss: Vol. 1. In: Attachment. New York: Basic Books, 1969.

[nsaf069-B8] Bowlby J. Attachment and loss: Vol. 2. In: Separation: Anxiety and Anger. New York: Basic Books, 1973.

[nsaf069-B9] Bradley MM , Lang PJ. Measuring emotion: the self-assessment manikin and the semantic differential. J Behav Ther Exp Psychiatry 1994;25:49–59. 10.1016/0005-7916(94)90063-9 7962581

[nsaf069-B10] Brennan KA , Clark CL, Shaver PR. Self-report measurement of adult attachment: an integrative overview. In: Simpson JA, Rholes WS(eds), Attachment Theory and Close Relationships. New York: Guilford Press, 1998, 46–76.

[nsaf069-B11] Buzsáki G , Draguhn A. Neuronal oscillations in cortical networks. Science 2004;304:1926–9. 10.1126/science.1099745 15218136

[nsaf069-B12] Carver CS , Johnson SL, Joormann J. Serotonergic function, two-mode models of self-regulation, and vulnerability to depression: what depression has in common with impulsive aggression. Psychol Bull 2008;134:912–43. 10.1037/a0013740 18954161 PMC2847478

[nsaf069-B13] Cassidy J. Emotion regulation: influences of attachment relationships. Monogr Soc Res Child Dev 1994;59:228–49. 7984163

[nsaf069-B14] Cassidy J , Shaver PR (eds). Handbook of Attachment: Theory, Research, and Clinical Applications. New York, NY: The Guilford Press, 2016.

[nsaf069-B15] Cavanagh JF , Frank MJ. Frontal theta as a mechanism for cognitive control. Trends Cogn Sci 2014;18:414–21. 10.1016/j.tics.2014.04.012 24835663 PMC4112145

[nsaf069-B16] Choi D , Sekiya T, Minote N et al Relative left frontal activity in reappraisal and suppression of negative emotion: evidence from frontal alpha asymmetry (FAA). Int J Psychophysiol 2016;109:37–44. 10.1016/j.ijpsycho.2016.09.018 27693504

[nsaf069-B17] Cludius B , Mennin D, Ehring T. Emotion regulation as a transdiagnostic process. Emotion 2020;20:37–42. 10.1037/emo0000646 31961175

[nsaf069-B18] Cohen MX , Cavanagh JF. Single-trial regression elucidates the role of prefrontal theta oscillations in response conflict. Front Psychol 2011;2:30. 10.3389/fpsyg.2011.00030 21713190 PMC3111011

[nsaf069-B19] Cools R , D'Esposito M. Inverted-U-shaped dopamine actions on human working memory and cognitive control. Biol Psychiatry 2011;69:e113–25. 10.1016/j.biopsych.2011.03.028 21531388 PMC3111448

[nsaf069-B20] Dayan P , Huys QJ. Serotonin in affective control. Annu Rev Neurosci 2009;32:95–126. 10.1146/annurev.neuro.051508.135607 19400722

[nsaf069-B21] De Cesarei A , Codispoti M. Affective modulation of the LPP and α-ERD during picture viewing. Psychophysiology 2011;48:1397–404. 10.1111/j.1469-8986.2011.01204.x 21486291

[nsaf069-B22] Del Popolo Cristaldi F , Mento G, Buodo G et al Emotion regulation strategies differentially modulate neural activity across affective prediction stages: an HD-EEG investigation. Front Behav Neurosci 2022;16:947063. 10.3389/fnbeh.2022.947063 35990725 PMC9388773

[nsaf069-B23] Delorme A , Makeig S. EEGLAB: an open source toolbox for analysis of single-trial EEG dynamics including independent component analysis. J Neurosci Methods 2004;134:9–21. 10.1016/j.jneumeth.2003.10.009 15102499

[nsaf069-B24] Dmochowski JP , Sajda P, Dias J et al Correlated components of ongoing EEG point to emotionally laden attention—a possible marker of engagement? Front Hum Neurosci 2012;6:112. 10.3389/fnhum.2012.00112 22623915 PMC3353265

[nsaf069-B25] Domic-Siede M , Guzmán-González M, Burgos J et al Emotion regulation strategies and the two-dimensional model of adult attachment: a pilot study. Front Behav Neurosci 2023a;17:1141607. 10.3389/fnbeh.2023.1141607 37484522 PMC10359990

[nsaf069-B26] Domic-Siede M , Guzmán-González M, Sánchez-Corzo A et al Emotion regulation unveiled through the categorical lens of attachment. BMC Psychol 2024b;12:240. 10.1186/s40359-024-01748-z 38678214 PMC11056069

[nsaf069-B27] Domic-Siede M , Guzmán-González M, Sánchez-Corzo A et al Pupil responses to emotion regulation strategies: the role of attachment orientations. Cogn Emot 2025b;1–18. 10.1080/02699931.2025.2512886 40472472

[nsaf069-B28] Domic-Siede M , Irani M, Valdés J et al Theta activity from frontopolar cortex, mid-cingulate cortex and anterior cingulate cortex shows different roles in cognitive planning performance. Neuroimage 2021;226:117557. 10.1016/j.neuroimage.2020.117557 33189934

[nsaf069-B29] Domic-Siede M , Irani M, Valdés J et al A visuospatial planning task coupled with eye-tracker and electroencephalogram systems. J Vis Exp 2023b;193:1–42. 10.3791/64622 36939245

[nsaf069-B30] Domic-Siede M , Sánchez-Corzo A, Álvarez X et al Human attachment and the electrophysiological dynamics of emotion regulation: an event-related potential study. Psychophysiology 2025a;62:e70075. 10.1111/psyp.70075 40395139

[nsaf069-B31] Domic-Siede M , Sánchez-Corzo A, Guzmán-González M. Brain oscillations during emotion regulation and the two-dimensional model of adult attachment. Biol Psychol 2024a;189:108793. 10.1016/j.biopsycho.2024.108793 38631550

[nsaf069-B32] Engel AK , Fries P. Beta-band oscillations—signalling the status quo? Curr Opin Neurobiol 2010;20:156–65. 10.1016/j.conb.2010.02.015 20359884

[nsaf069-B33] Ertl M , Hildebrandt M, Ourina K et al Emotion regulation by cognitive reappraisal—the role of frontal theta oscillations. Neuroimage 2013;81:412–21. 10.1016/j.neuroimage.2013.05.044 23689018

[nsaf069-B34] *Faul F, Erdfelder E, Lang AG et al GPower 3: a flexible statistical power analysis program for the social, behavioral, and biomedical sciences. Behav Res Methods 2007;39:175–91. 10.3758/bf03193146 17695343

[nsaf069-B35] Field A , 2013. Discovering Statistics Using IBM SPSS Statistics (4th ed.) . Thousand Oaks, CA: SAGE Publications.

[nsaf069-B36] Gilbertson T , Lalo E, Doyle L et al Existing motor state is favored at the expense of new movement during 13–35 Hz oscillatory synchrony in the human corticospinal system. J Neurosci 2005;25:7771–9. 10.1523/JNEUROSCI.1762-05.2005 16120778 PMC6725263

[nsaf069-B37] Grech R , Cassar T, Muscat J et al Review on solving the inverse problem in EEG source analysis. J Neuroeng Rehabil 2008;5:25. 10.1186/1743-0003-5-25 18990257 PMC2605581

[nsaf069-B38] Gross JJ. The emerging field of emotion regulation: an integrative review. Rev Gen Psychol 1998;2:271–99. 10.1037/1089-2680.2.3.271

[nsaf069-B39] Gross J. Emotion regulation in adulthood: timing is everything. Curr Dir Psychol Sci 2001;10:214–9. 10.1111/1467-8721.00152

[nsaf069-B40] Gross JJ. Emotion regulation: affective, cognitive, and social consequences. Psychophysiology 2002;39:281–91. 10.1017/s0048577201393198 12212647

[nsaf069-B41] Gross JJ. Emotion regulation: current status and future prospects. Psychol Inq 2015;26:1–26. 10.1080/1047840X.2014.940781

[nsaf069-B42] Gross JJ. Emotion regulation: conceptual and empirical foundations. In: Gross JJ, Ford BQ (eds), Handbook of Emotion Regulation, 3rd ed. New York, NY: Guilford Press, 2024, 3–12.

[nsaf069-B43] Gross JJ , John OP. Individual differences in two emotion regulation processes: implications for affect, relationships, and well-being. J Pers Soc Psychol 2003;85:348–62. 10.1037/0022-3514.85.2.348 12916575

[nsaf069-B44] Guzmán-González M , Calderón C, Domic-Siede M et al Propuesta de valores de referencia Para el cuestionario de evaluación del apego adulto: Experiences in close relationships (ECR-12), en población adulta chilena. Ter Psicol 2023;41:39–61. 10.4067/S0718-48082023000100039

[nsaf069-B45] Guzmán-González M , Rivera-Ottenberger D, Brassard A et al Measuring adult romantic attachment: psychometric properties of the brief Spanish version of the experiences in close relationships. Psicol Reflex Crit 2020;33:9. 10.1186/s41155-020-00145-w 32542456 PMC7295914

[nsaf069-B46] Hajcak G , Nieuwenhuis S. Reappraisal modulates the electrocortical response to unpleasant pictures. Cogn Affect Behav Neurosci 2006;6:291–7. 10.3758/CABN.6.4.291 17458444

[nsaf069-B47] Heinrichs-Graham E , Wilson TW. Is an absolute level of cortical beta suppression required for proper movement? Neuroimage 2016;134:514–21. 10.1016/j.neuroimage.2016.04.032 27090351 PMC4912897

[nsaf069-B48] Jung TP , Makeig S, Humphries C et al Removing electroencephalographic artifacts by blind source separation. Psychophysiology 2000;37:163–78. 10.1111/1469-8986.3720163 10731767

[nsaf069-B49] Keil A , Debener S, Gratton G et al Committee report: publication guidelines and recommendations for studies using electroencephalography and magnetoencephalography. Psychophysiology 2014;51:1–21. 10.1111/psyp.12147 24147581

[nsaf069-B50] Koval P , Kalokerinos EK. Daily diaries and ecological momentary assessment. In: Gross JJ (ed.), Handbook of Emotion Regulation, 3rd ed. New York, NY: Guilford Press, 2024, 31–49.

[nsaf069-B51] Lai M , Demuru M, Hillebrand A et al A comparison between scalp- and source-reconstructed EEG networks. Sci Rep 2018;8:12269. 10.1038/s41598-018-30869-w 30115955 PMC6095906

[nsaf069-B52] Lang PJ , Bradley MM, Cuthbert BN. International Affective Picture System (IAPS): Instruction Manual and Affective Ratings Technical Report A6. Gainesville, FL: University of Florida, 2005.

[nsaf069-B53] Lapomarda G , Valer S, Job R et al Built to last: Theta and Delta changes in resting-state EEG activity after regulating emotions. Brain Behav 2022;12:e2597. 10.1002/brb3.2597 35560984 PMC9226824

[nsaf069-B54] Li W , Zhang W, Jiang Z et al Source localization and functional network analysis in emotion cognitive reappraisal with EEG-fMRI integration. Front Hum Neurosci 2022;16:960784. 10.3389/fnhum.2022.960784 36034109 PMC9411793

[nsaf069-B55] Long M , Verbeke W, Ein-Dor T et al A functional neuro-anatomical model of human attachment (NAMA): insights from first- and second-person social neuroscience. Cortex 2020;126:281–321. 10.1016/j.cortex.2020.01.010 32092496

[nsaf069-B56] MacNamara A , Joyner K, Klawohn J. Event-related potential studies of emotion regulation: a review of recent progress and future directions. Int J Psychophysiol 2022;176:73–88. 10.1016/j.ijpsycho.2022.03.008 35346736 PMC9081270

[nsaf069-B57] Matuschek H , Kliegl R, Vasishth S et al Balancing type I error and power in linear mixed models. J Mem Lang 2017;94:305–15. 10.1016/j.jml.2017.01.001

[nsaf069-B58] Michel CM , Brunet D. EEG source imaging: a practical review of the analysis steps. Front Neurol 2019;10:325. 10.3389/fneur.2019.00325 31019487 PMC6458265

[nsaf069-B59] Mikulincer M , Shaver PR, 2007. Attachment in Adulthood: Structure, Dynamics, and Change. New York, NY: Guilford Press.

[nsaf069-B60] Mikulincer M , Shaver P, 2016. Attachment in Adulthood: Structure, Dynamics, and Change, 2nd ed. New York, NY: Guilford Publications.

[nsaf069-B61] Mikulincer M , Shaver PR. Attachment orientations and emotion regulation. Curr Opin Psychol 2019;25:6–10. 10.1016/j.copsyc.2018.02.006 29494853

[nsaf069-B62] Mishra P , Pandey CM, Singh U et al Descriptive statistics and normality tests for statistical data. Ann Card Anaesth 2019;22:67–72. 10.4103/aca.ACA_157_18 30648682 PMC6350423

[nsaf069-B63] Moser JS , Hajcak G, Bukay E et al Intentional modulation of emotional responding to unpleasant pictures: an ERP study. Psychophysiology 2006;43:292–6. 10.1111/j.1469-8986.2006.00402.x 16805868

[nsaf069-B64] Moser JS , Krompinger JW, Dietz J et al Electrophysiological correlates of decreasing and increasing emotional responses to unpleasant pictures. Psychophysiology 2009;46:17–27. 10.1111/j.1469-8986.2008.00721.x 18992073

[nsaf069-B65] Murata A , Moser JS, Kitayama S. Culture shapes electrocortical responses during emotion suppression. Soc Cogn Affect Neurosci 2013;8:595–601. 10.1093/scan/nss036 22422803 PMC3682443

[nsaf069-B66] Ochsner KN , Ray RD, Cooper JC et al For better or for worse: neural systems supporting the cognitive down- and up-regulation of negative emotion. Neuroimage 2004;23:483–99. 10.1016/j.neuroimage.2004.06.030 15488398

[nsaf069-B67] Oostenveld R , Fries P, Maris E et al FieldTrip: open source software for advanced analysis of MEG, EEG, and invasive electrophysiological data. Comput Intell Neurosci 2011;2011:156869. 10.1155/2011/156869 21253357 PMC3021840

[nsaf069-B68] Papousek I , Weiss EM, Perchtold CM et al The capacity for generating cognitive reappraisals is reflected in asymmetric activation of frontal brain regions. Brain Imaging Behav 2017;11:577–90. 10.1007/s11682-016-9537-2 26935554 PMC5408052

[nsaf069-B69] Pion-Tonachini L , Kreutz-Delgado K, Makeig S. ICLabel: an automated electroencephalographic independent component classifier, dataset, and website. Neuroimage 2019;198:181–97. 10.1016/j.neuroimage.2019.05.026 31103785 PMC6592775

[nsaf069-B70] Pizzagalli DA. Depression, stress, and anhedonia: toward a synthesis and integrated model. Annu Rev Clin Psychol 2014;10:393–423. 10.1146/annurev-clinpsy-050212-185606 24471371 PMC3972338

[nsaf069-B71] Ramos-Henderson M , Guzmán-González M, Bahamondes J et al The moderating role of the late positive potential in the link between attachment anxiety and emotion regulation difficulties. Front Psychol 2024;15:1360366. 10.3389/fpsyg.2024.1360366 39606193 PMC11598532

[nsaf069-B72] Sadaghiani S , Kleinschmidt A. Brain networks and alpha oscillations: structural and functional foundations of cognitive control. Trends Cogn Sci 2016;20:805–17. 10.1016/j.tics.2016.09.004 27707588

[nsaf069-B73] Schlumpf YR , Nijenhuis ER, Klein C et al Functional reorganization of neural networks involved in emotion regulation following trauma therapy for complex trauma disorders. Neuroimage Clin 2019;23:101807. 10.1016/j.nicl.2019.101807 30986752 PMC6505069

[nsaf069-B74] Shackman AJ , Salomons TV, Slagter HA et al The integration of negative affect, pain and cognitive control in the cingulate cortex. Nat Rev Neurosci 2011;12:154–67. 10.1038/nrn2994 21331082 PMC3044650

[nsaf069-B75] Shaver PR , Mikulincer M. Attachment-related psychodynamics. Attach Hum Dev 2002;4:133–61. 10.1080/14616730210154171 12467506

[nsaf069-B76] Soto JA , Perez CR, Kim YH et al Is expressive suppression always associated with poorer psychological functioning? A cross-cultural comparison between European Americans and Hong Kong Chinese. Emotion 2011;11:1450–5. 10.1037/a0023340 21707152

[nsaf069-B77] Spencer R , Guzmán M, Fresno A et al Validación chilena del cuestionario de evaluación del apego romántico experiences in close relationships (ECR): análisis de la validez de criterio. Ter Psicol 2013;31:313–24. 10.4067/s0718-48082013000300006

[nsaf069-B78] Sulpizio S , Grecucci A, Job R. Tune in to the right frequency: Theta changes when distancing from emotions elicited by unpleasant images and words. Eur J Neurosci 2021;53:916–28. 10.1111/ejn.15013 33091188

[nsaf069-B79] Swann N , Tandon N, Canolty R et al Intracranial EEG reveals a time- and frequency-specific role for the right inferior frontal gyrus and primary motor cortex in stopping initiated responses. J Neurosci 2009;29:12675–85. 10.1523/JNEUROSCI.3359-09.2009 19812342 PMC2801605

[nsaf069-B80] Uusberg A , Thiruchselvam R, Gross JJ. Using distraction to regulate emotion: insights from EEG theta dynamics. Int J Psychophysiol 2014;91:254–60. 10.1016/j.ijpsycho.2014.01.006 24440597

[nsaf069-B81] Vinck M , Oostenveld R, van Wingerden M et al An improved index of phase-synchronization for electrophysiological data in the presence of volume-conduction, noise and sample-size bias. Neuroimage 2011;55:1548–65. 10.1016/j.neuroimage.2011.01.055 21276857

[nsaf069-B82] Vrtička P , Bondolfi G, Sander D et al The neural substrates of social emotion perception and regulation are modulated by adult attachment style. Soc Neurosci 2012;7:473–93. 10.1080/17470919.2011.647410 22217336

[nsaf069-B83] Vrtička P , Vuilleumier P. Neuroscience of human social interactions and adult attachment style. Front Hum Neurosci 2012;6:212– 10.3389/fnhum.2012.00212 22822396 PMC3398354

[nsaf069-B84] Wei L , Zhao M, Yang X, Li Y, 2017. Theta oscillations during cognitive reappraisal of sad and fearful stimuli. In: *2017 8th International IEEE/EMBS Conference on Neural Engineering (NER)*, 560–563. 10.1109/NER.2017.8008413

[nsaf069-B85] Womelsdorf T , Johnston K, Vinck M et al Theta-activity in anterior cingulate cortex predicts task rules and their adjustments following errors. Proc Natl Acad Sci USA 2010;107:5248–53. 10.1073/pnas.0906194107 20194767 PMC2841867

[nsaf069-B86] Yan C , Ding Q, Wang Y et al The effect of cognitive reappraisal and expression suppression on sadness and the recognition of sad scenes: an event-related potential study. Front Psychol 2022;13:935007. 10.3389/fpsyg.2022.935007 36211892 PMC9537681

[nsaf069-B87] Yuan J , Long Q, Ding N et al Suppression dampens unpleasant emotion faster than reappraisal: neural dynamics in a Chinese sample. Sci China Life Sci 2015;58:480–91. 10.1007/s11427-014-4739-6 25316046

[nsaf069-B88] Zouaoui I , Zellag M, Hernout J et al Alpha and theta oscillations during the cognitive reappraisal of aversive pictures: a spatio-temporal qEEG investigation. Int J Psychophysiol 2023;192:13–25. 10.1016/j.ijpsycho.2023.07.001 37490956

