# Supplementary material for: Phase-synchronized brain connectivity during emotion regulation: attachment as a moderator
Source: Soc Cogn Affect Neurosci. 2025 Jul 1;20(1):nsaf069. doi: 10.1093/scan/nsaf069 (PMC12398388; doi:10.1093/scan/nsaf069)
Supplement: nsaf069_Supplementary_Data [file nsaf069_supplementary_data.docx]

**Supplementary Materials**

**Supplementary Table S1. Selected IAPS pictures per condition (Lang et al., 2005)**

|  | Experimental Conditions | | | |
| --- | --- | --- | --- | --- |
| N° | Nat-negative | Suppress | Reappraise | Nat-neutral |
| 1 | 2683 | 2095 | 2130 | 7009 |
| 2 | 2703 | 2205 | 2700 | 7011 |
| 3 | 2710 | 2900 | 2751 | 7012 |
| 4 | 2717 | 6211 | 2811 | 7018 |
| 5 | 3220 | 6231 | 3550 | 7042 |
| 6 | 3230 | 6250 | 6260 | 7045 |
| 7 | 3500 | 6312 | 6350 | 7061 |
| 8 | 6300 | 6560 | 6530 | 2190 |
| 9 | 6315 | 6563 | 6561 | 2215 |
| 10 | 6360 | 6838 | 6562 | 2359 |
| 11 | 6540 | 9041 | 6825 | 2499 |
| 12 | 6550 | 9250 | 6834 | 2518 |
| 13 | 6840 | 9421 | 9050 | 2521 |
| 14 | 9429 | 9427 | 9419 | 2593 |
| 15 | 9900 | 9435 | 9425 | 2594 |

Nat-negative: Natural condition containing negative valence pictures; Nat-neutral: Natural condition containing neutral valence pictures.

**Supplementary Table S2. Descriptive statistics of the selected IAPS pictures per condition.**

|  | Arousal | |
| --- | --- | --- |
|  | Mean | SD |
| Nat-negative | 6.09 | 0.59 |
| Suppress | 5.76 | 0.81 |
| Reappraise | 5.83 | 0.81 |
| Nat-neutral | 3.48 | 0.48 |

Nat-negative: Natural condition containing negative valence pictures; Nat-neutral: Natural condition containing neutral valence pictures. SD = Standard Deviation.

**Supplementary Table S3.** **Multiple comparison test for the arousal of IAPS pictures between conditions**

| Tukey’s test | Mean Diff. | 95.00% CI of diff. | Summary | Adjusted p-value |
| --- | --- | --- | --- | --- |
| Natural-Neg vs. Suppress | 0.3240 | -0.3415 to 0.9895 | ns | 0.5736 |
| Natural-Neg vs. Reappraise | 0.2633 | -0.4022 to 0.9288 | ns | 0.7222 |
| **Natural-Neg vs. Natural-Neu** | **2.607** | **1.942 to 3.273** | ******** | **<0.0001** |
| Suppress vs. Reappraise | -0.06067 | -0.7262 to 0.6048 | ns | 0.9950 |
| **Suppress vs. Natural-Neu** | **2.283** | **1.618 to 2.949** | ******** | **<0.0001** |
| **Reappraise vs. Natural-Neu** | **2.344** | **1.679 to 3.009** | ******** | **<0.0001** |

Natural-neg: Natural condition containing negative valence pictures; Natural-neu: Natural condition containing neutral valence pictures.; ns: not significant.

**Supplementary Table S4. Electrode Pairs Included in the Frontal and the Central ROI for Each Frequency Band**

| **Electrode Pairs Frontal ROI (16 electrodes):**  roi_frontal = {'Fp1','Fp2','Fpz','Fz','F3','F4','F7','F8','AF3','AF4','AF7','AF8','FC1','FC2','FC5','FC6'}; | | | |
| --- | --- | --- | --- |
| **Delta (246 pairs)** | **Theta (237 pairs)** | **Alpha (273 pairs)** | **Beta (266 pairs)** |
| 'F3 - C3'  'F3 - C4'  'F3 - CP1'  'F3 - CP2'  'F3 - CP6'  'F3 - Cz'  'F3 - F4'  'F3 - F8'  'F3 - FC1'  'F3 - FC2'  'F3 - FC5'  'F3 - FC6'  'F3 - Fz'  'F3 - O1'  'F3 - O2'  'F3 - Oz'  'F3 - P4'  'F3 - P8'  'F3 - POz'  'F3 - Pz'  'F3 - T7'  'F3 - T8'  'F4 - C3'  'F4 - C4'  'F4 - CP1'  'F4 - CP2'  'F4 - CP5'  'F4 - CP6'  'F4 - Cz'  'F4 - F8'  'F4 - FC1'  'F4 - FC2'  'F4 - FC5'  'F4 - FC6'  'F4 - O1'  'F4 - O2'  'F4 - Oz'  'F4 - P3'  'F4 - P4'  'F4 - P7'  'F4 - P8'  'F4 - POz'  'F4 - Pz'  'F4 - T7'  'F4 - T8'  'F7 - C3'  'F7 - C4'  'F7 - CP1'  'F7 - CP2'  'F7 - CP5'  'F7 - CP6'  'F7 - Cz'  'F7 - F3'  'F7 - F4'  'F7 - F8'  'F7 - FC1'  'F7 - FC2'  'F7 - FC5'  'F7 - FC6'  'F7 - Fz'  'F7 - O1'  'F7 - O2'  'F7 - Oz'  'F7 - P3'  'F7 - P4'  'F7 - P8'  'F7 - POz'  'F7 - Pz'  'F7 - T8'  'F8 - C3'  'F8 - CP1'  'F8 - CP5'  'F8 - Cz'  'F8 - FC1'  'F8 - FC2'  'F8 - FC5'  'F8 - FC6'  'F8 - O1'  'F8 - O2'  'F8 - Oz'  'F8 - P3'  'F8 - P4'  'F8 - P7'  'F8 - P8'  'F8 - POz'  'F8 - Pz'  'F8 - T7'  'F8 - T8'  'FC1 - C3'  'FC1 - C4'  'FC1 - CP1'  'FC1 - CP2'  'FC1 - Cz'  'FC1 - FC2'  'FC1 - FC6'  'FC1 - O1'  'FC1 - O2'  'FC1 - Oz'  'FC1 - P4'  'FC1 - P7'  'FC1 - P8'  'FC1 - POz'  'FC1 - Pz'  'FC1 - T7'  'FC1 - T8'  'FC2 - C3'  'FC2 - C4'  'FC2 - CP1'  'FC2 - CP2'  'FC2 - CP5'  'FC2 - CP6'  'FC2 - Cz'  'FC2 - FC6'  'FC2 - O1'  'FC2 - O2'  'FC2 - Oz'  'FC2 - P3'  'FC2 - P4'  'FC2 - P7'  'FC2 - P8'  'FC2 - POz'  'FC2 - Pz'  'FC2 - T7'  'FC2 - T8'  'FC5 - C3'  'FC5 - C4'  'FC5 - CP1'  'FC5 - CP2'  'FC5 - CP5'  'FC5 - Cz'  'FC5 - FC1'  'FC5 - FC2'  'FC5 - O1'  'FC5 - O2'  'FC5 - Oz'  'FC5 - P3'  'FC5 - P4'  'FC5 - P8'  'FC5 - POz'  'FC5 - Pz'  'FC5 - T7'  'FC5 - T8'  'FC6 - C3'  'FC6 - C4'  'FC6 - CP1'  'FC6 - CP2'  'FC6 - CP5'  'FC6 - Cz'  'FC6 - O1'  'FC6 - O2'  'FC6 - Oz'  'FC6 - P3'  'FC6 - P4'  'FC6 - P7'  'FC6 - P8'  'FC6 - POz'  'FC6 - Pz'  'FC6 - T7'  'FC6 - T8'  'Fp1 - C3'  'Fp1 - C4'  'Fp1 - CP1'  'Fp1 - CP2'  'Fp1 - CP6'  'Fp1 - Cz'  'Fp1 - F3'  'Fp1 - F4'  'Fp1 - F7'  'Fp1 - F8'  'Fp1 - FC1'  'Fp1 - FC2'  'Fp1 - FC5'  'Fp1 - FC6'  'Fp1 - Fp2'  'Fp1 - Fpz'  'Fp1 - Fz'  'Fp1 - O1'  'Fp1 - O2'  'Fp1 - Oz'  'Fp1 - P3'  'Fp1 - P4'  'Fp1 - P8'  'Fp1 - POz'  'Fp1 - Pz'  'Fp1 - T8'  'Fp2 - C3'  'Fp2 - C4'  'Fp2 - CP1'  'Fp2 - CP2'  'Fp2 - CP6'  'Fp2 - Cz'  'Fp2 - F3'  'Fp2 - F7'  'Fp2 - F8'  'Fp2 - FC1'  'Fp2 - FC2'  'Fp2 - FC5'  'Fp2 - Fz'  'Fp2 - O1'  'Fp2 - O2'  'Fp2 - Oz'  'Fp2 - P4'  'Fp2 - P8'  'Fp2 - POz'  'Fp2 - Pz'  'Fp2 - T7'  'Fp2 - T8'  'Fpz - C3'  'Fpz - C4'  'Fpz - CP1'  'Fpz - CP2'  'Fpz - Cz'  'Fpz - F3'  'Fpz - F7'  'Fpz - F8'  'Fpz - FC1'  'Fpz - FC2'  'Fpz - FC5'  'Fpz - Fp2'  'Fpz - Fz'  'Fpz - O1'  'Fpz - O2'  'Fpz - Oz'  'Fpz - P4'  'Fpz - P8'  'Fpz - POz'  'Fpz - Pz'  'Fz - C4'  'Fz - CP1'  'Fz - CP2'  'Fz - CP6'  'Fz - Cz'  'Fz - F4'  'Fz - F8'  'Fz - FC2'  'Fz - FC5'  'Fz - FC6'  'Fz - O1'  'Fz - O2'  'Fz - Oz'  'Fz - P4'  'Fz - P7'  'Fz - P8'  'Fz - POz'  'Fz - Pz'  'Fz - T7' | 'F3 - C4'  'F3 - CP2'  'F3 - CP5'  'F3 - CP6'  'F3 - Cz'  'F3 - F4'  'F3 - F8'  'F3 - FC1'  'F3 - FC2'  'F3 - FC5'  'F3 - FC6'  'F3 - Fz'  'F3 - O1'  'F3 - O2'  'F3 - Oz'  'F3 - P3'  'F3 - P4'  'F3 - P7'  'F3 - P8'  'F3 - POz'  'F3 - Pz'  'F3 - T7'  'F3 - T8'  'F4 - C3'  'F4 - C4'  'F4 - CP1'  'F4 - CP2'  'F4 - CP5'  'F4 - CP6'  'F4 - Cz'  'F4 - F8'  'F4 - FC2'  'F4 - FC5'  'F4 - FC6'  'F4 - O1'  'F4 - O2'  'F4 - Oz'  'F4 - P3'  'F4 - P4'  'F4 - P7'  'F4 - P8'  'F4 - POz'  'F4 - Pz'  'F4 - T8'  'F7 - C3'  'F7 - C4'  'F7 - CP1'  'F7 - CP2'  'F7 - CP5'  'F7 - CP6'  'F7 - Cz'  'F7 - F4'  'F7 - FC1'  'F7 - FC2'  'F7 - FC5'  'F7 - FC6'  'F7 - Fz'  'F7 - O1'  'F7 - O2'  'F7 - Oz'  'F7 - P3'  'F7 - P4'  'F7 - P7'  'F7 - P8'  'F7 - POz'  'F7 - Pz'  'F7 - T7'  'F7 - T8'  'F8 - C3'  'F8 - C4'  'F8 - CP1'  'F8 - CP2'  'F8 - CP5'  'F8 - CP6'  'F8 - Cz'  'F8 - FC1'  'F8 - FC2'  'F8 - FC6'  'F8 - O1'  'F8 - O2'  'F8 - P3'  'F8 - P8'  'F8 - POz'  'F8 - Pz'  'F8 - T8'  'FC1 - C3'  'FC1 - C4'  'FC1 - CP2'  'FC1 - CP5'  'FC1 - CP6'  'FC1 - Cz'  'FC1 - FC6'  'FC1 - O1'  'FC1 - O2'  'FC1 - Oz'  'FC1 - P3'  'FC1 - P4'  'FC1 - P7'  'FC1 - P8'  'FC1 - POz'  'FC1 - Pz'  'FC1 - T7'  'FC1 - T8'  'FC2 - C3'  'FC2 - C4'  'FC2 - CP1'  'FC2 - CP2'  'FC2 - CP5'  'FC2 - CP6'  'FC2 - Cz'  'FC2 - FC6'  'FC2 - O1'  'FC2 - O2'  'FC2 - Oz'  'FC2 - P3'  'FC2 - P4'  'FC2 - P7'  'FC2 - P8'  'FC2 - POz'  'FC2 - Pz'  'FC2 - T7'  'FC2 - T8'  'FC5 - C3'  'FC5 - C4'  'FC5 - CP1'  'FC5 - CP2'  'FC5 - CP5'  'FC5 - CP6'  'FC5 - Cz'  'FC5 - FC1'  'FC5 - FC2'  'FC5 - O1'  'FC5 - O2'  'FC5 - Oz'  'FC5 - P3'  'FC5 - P4'  'FC5 - P7'  'FC5 - P8'  'FC5 - POz'  'FC5 - Pz'  'FC5 - T7'  'FC5 - T8'  'FC6 - C3'  'FC6 - C4'  'FC6 - CP1'  'FC6 - CP2'  'FC6 - CP5'  'FC6 - CP6'  'FC6 - Cz'  'FC6 - O1'  'FC6 - O2'  'FC6 - Oz'  'FC6 - P3'  'FC6 - P4'  'FC6 - P8'  'FC6 - POz'  'FC6 - Pz'  'FC6 - T8'  'Fp1 - C4'  'Fp1 - CP2'  'Fp1 - CP6'  'Fp1 - Cz'  'Fp1 - F3'  'Fp1 - F8'  'Fp1 - FC1'  'Fp1 - FC2'  'Fp1 - FC6'  'Fp1 - O1'  'Fp1 - O2'  'Fp1 - Oz'  'Fp1 - P3'  'Fp1 - P4'  'Fp1 - P7'  'Fp1 - P8'  'Fp1 - POz'  'Fp1 - Pz'  'Fp1 - T8'  'Fp2 - C4'  'Fp2 - CP2'  'Fp2 - CP6'  'Fp2 - Cz'  'Fp2 - F8'  'Fp2 - FC1'  'Fp2 - FC2'  'Fp2 - FC6'  'Fp2 - Fz'  'Fp2 - O1'  'Fp2 - O2'  'Fp2 - Oz'  'Fp2 - P4'  'Fp2 - P8'  'Fp2 - POz'  'Fp2 - Pz'  'Fp2 - T8'  'Fpz - C4'  'Fpz - CP2'  'Fpz - CP5'  'Fpz - CP6'  'Fpz - Cz'  'Fpz - F3'  'Fpz - F4'  'Fpz - F7'  'Fpz - F8'  'Fpz - FC1'  'Fpz - FC2'  'Fpz - FC6'  'Fpz - Fp2'  'Fpz - Fz'  'Fpz - O1'  'Fpz - O2'  'Fpz - Oz'  'Fpz - P4'  'Fpz - P8'  'Fpz - POz'  'Fpz - Pz'  'Fpz - T8'  'Fz - C4'  'Fz - CP2'  'Fz - CP5'  'Fz - CP6'  'Fz - Cz'  'Fz - F4'  'Fz - F8'  'Fz - FC1'  'Fz - FC5'  'Fz - FC6'  'Fz - O1'  'Fz - O2'  'Fz - Oz'  'Fz - P3'  'Fz - P4'  'Fz - P7'  'Fz - P8'  'Fz - POz'  'Fz - Pz'  'Fz - T7'  'Fz - T8' | 'F3 - C3'  'F3 - C4'  'F3 - CP1'  'F3 - CP2'  'F3 - CP5'  'F3 - CP6'  'F3 - Cz'  'F3 - F4'  'F3 - F8'  'F3 - FC1'  'F3 - FC2'  'F3 - FC5'  'F3 - FC6'  'F3 - Fz'  'F3 - O1'  'F3 - O2'  'F3 - Oz'  'F3 - P3'  'F3 - P4'  'F3 - P7'  'F3 - P8'  'F3 - POz'  'F3 - Pz'  'F3 - T7'  'F3 - T8'  'F4 - C3'  'F4 - C4'  'F4 - CP1'  'F4 - CP2'  'F4 - CP5'  'F4 - CP6'  'F4 - Cz'  'F4 - F8'  'F4 - FC1'  'F4 - FC2'  'F4 - FC5'  'F4 - FC6'  'F4 - O1'  'F4 - O2'  'F4 - Oz'  'F4 - P3'  'F4 - P4'  'F4 - P7'  'F4 - POz'  'F4 - Pz'  'F4 - T7'  'F4 - T8'  'F7 - C3'  'F7 - C4'  'F7 - CP1'  'F7 - CP2'  'F7 - CP5'  'F7 - CP6'  'F7 - Cz'  'F7 - F3'  'F7 - F4'  'F7 - F8'  'F7 - FC1'  'F7 - FC2'  'F7 - FC5'  'F7 - FC6'  'F7 - Fz'  'F7 - O1'  'F7 - O2'  'F7 - Oz'  'F7 - P3'  'F7 - P4'  'F7 - P8'  'F7 - POz'  'F7 - Pz'  'F7 - T7'  'F7 - T8'  'F8 - C3'  'F8 - C4'  'F8 - CP1'  'F8 - CP2'  'F8 - CP5'  'F8 - Cz'  'F8 - FC1'  'F8 - FC2'  'F8 - FC5'  'F8 - FC6'  'F8 - O2'  'F8 - Oz'  'F8 - P3'  'F8 - P4'  'F8 - P8'  'F8 - POz'  'F8 - Pz'  'F8 - T7'  'F8 - T8'  'FC1 - C3'  'FC1 - C4'  'FC1 - CP1'  'FC1 - CP2'  'FC1 - CP5'  'FC1 - CP6'  'FC1 - Cz'  'FC1 - FC2'  'FC1 - FC6'  'FC1 - O1'  'FC1 - O2'  'FC1 - Oz'  'FC1 - P3'  'FC1 - P4'  'FC1 - P7'  'FC1 - P8'  'FC1 - POz'  'FC1 - Pz'  'FC1 - T7'  'FC1 - T8'  'FC2 - C3'  'FC2 - C4'  'FC2 - CP1'  'FC2 - CP2'  'FC2 - CP5'  'FC2 - CP6'  'FC2 - Cz'  'FC2 - FC6'  'FC2 - O1'  'FC2 - O2'  'FC2 - Oz'  'FC2 - P3'  'FC2 - P4'  'FC2 - P7'  'FC2 - P8'  'FC2 - POz'  'FC2 - Pz'  'FC2 - T7'  'FC2 - T8'  'FC5 - C4'  'FC5 - CP1'  'FC5 - CP2'  'FC5 - CP5'  'FC5 - CP6'  'FC5 - Cz'  'FC5 - FC1'  'FC5 - FC2'  'FC5 - FC6'  'FC5 - O1'  'FC5 - O2'  'FC5 - Oz'  'FC5 - P3'  'FC5 - P4'  'FC5 - P7'  'FC5 - P8'  'FC5 - POz'  'FC5 - Pz'  'FC5 - T7'  'FC5 - T8'  'FC6 - C3'  'FC6 - C4'  'FC6 - CP1'  'FC6 - CP2'  'FC6 - CP5'  'FC6 - CP6'  'FC6 - Cz'  'FC6 - O1'  'FC6 - Oz'  'FC6 - P3'  'FC6 - P4'  'FC6 - P7'  'FC6 - P8'  'FC6 - POz'  'FC6 - Pz'  'FC6 - T7'  'FC6 - T8'  'Fp1 - C3'  'Fp1 - C4'  'Fp1 - CP1'  'Fp1 - CP2'  'Fp1 - CP5'  'Fp1 - CP6'  'Fp1 - Cz'  'Fp1 - F3'  'Fp1 - F4'  'Fp1 - F7'  'Fp1 - F8'  'Fp1 - FC1'  'Fp1 - FC2'  'Fp1 - FC5'  'Fp1 - FC6'  'Fp1 - Fp2'  'Fp1 - Fpz'  'Fp1 - Fz'  'Fp1 - O1'  'Fp1 - O2'  'Fp1 - Oz'  'Fp1 - P3'  'Fp1 - P4'  'Fp1 - P7'  'Fp1 - POz'  'Fp1 - Pz'  'Fp1 - T7'  'Fp1 - T8'  'Fp2 - C3'  'Fp2 - C4'  'Fp2 - CP1'  'Fp2 - CP2'  'Fp2 - CP5'  'Fp2 - CP6'  'Fp2 - Cz'  'Fp2 - F3'  'Fp2 - F4'  'Fp2 - F7'  'Fp2 - F8'  'Fp2 - FC1'  'Fp2 - FC2'  'Fp2 - FC5'  'Fp2 - FC6'  'Fp2 - Fz'  'Fp2 - O1'  'Fp2 - O2'  'Fp2 - Oz'  'Fp2 - P3'  'Fp2 - P4'  'Fp2 - P7'  'Fp2 - P8'  'Fp2 - POz'  'Fp2 - Pz'  'Fp2 - T7'  'Fp2 - T8'  'Fpz - C3'  'Fpz - C4'  'Fpz - CP1'  'Fpz - CP2'  'Fpz - CP5'  'Fpz - CP6'  'Fpz - Cz'  'Fpz - F3'  'Fpz - F4'  'Fpz - F7'  'Fpz - F8'  'Fpz - FC1'  'Fpz - FC2'  'Fpz - FC5'  'Fpz - FC6'  'Fpz - Fp2'  'Fpz - Fz'  'Fpz - O1'  'Fpz - O2'  'Fpz - Oz'  'Fpz - P3'  'Fpz - P4'  'Fpz - P7'  'Fpz - POz'  'Fpz - Pz'  'Fpz - T7'  'Fpz - T8'  'Fz - C3'  'Fz - C4'  'Fz - CP1'  'Fz - CP2'  'Fz - CP5'  'Fz - CP6'  'Fz - Cz'  'Fz - F4'  'Fz - F8'  'Fz - FC1'  'Fz - FC2'  'Fz - FC5'  'Fz - FC6'  'Fz - O1'  'Fz - O2'  'Fz - Oz'  'Fz - P3'  'Fz - P4'  'Fz - P7'  'Fz - P8'  'Fz - POz'  'Fz - Pz'  'Fz - T7'  'Fz - T8' | 'F3 - C3'  'F3 - C4'  'F3 - CP1'  'F3 - CP5'  'F3 - CP6'  'F3 - Cz'  'F3 - F4'  'F3 - F8'  'F3 - FC1'  'F3 - FC2'  'F3 - FC5'  'F3 - FC6'  'F3 - Fz'  'F3 - O1'  'F3 - O2'  'F3 - Oz'  'F3 - P3'  'F3 - P4'  'F3 - P7'  'F3 - P8'  'F3 - POz'  'F3 - Pz'  'F3 - T7'  'F3 - T8'  'F4 - C3'  'F4 - CP1'  'F4 - CP2'  'F4 - CP5'  'F4 - CP6'  'F4 - Cz'  'F4 - F8'  'F4 - FC1'  'F4 - FC2'  'F4 - FC5'  'F4 - FC6'  'F4 - O1'  'F4 - O2'  'F4 - Oz'  'F4 - P3'  'F4 - P4'  'F4 - P7'  'F4 - P8'  'F4 - POz'  'F4 - Pz'  'F4 - T7'  'F4 - T8'  'F7 - C3'  'F7 - C4'  'F7 - CP1'  'F7 - CP2'  'F7 - CP5'  'F7 - CP6'  'F7 - Cz'  'F7 - F3'  'F7 - F4'  'F7 - F8'  'F7 - FC1'  'F7 - FC5'  'F7 - FC6'  'F7 - Fz'  'F7 - O1'  'F7 - O2'  'F7 - Oz'  'F7 - P3'  'F7 - P4'  'F7 - P7'  'F7 - P8'  'F7 - POz'  'F7 - Pz'  'F7 - T7'  'F7 - T8'  'F8 - C3'  'F8 - C4'  'F8 - CP1'  'F8 - CP2'  'F8 - CP5'  'F8 - CP6'  'F8 - Cz'  'F8 - FC1'  'F8 - FC2'  'F8 - FC5'  'F8 - FC6'  'F8 - O1'  'F8 - P3'  'F8 - P7'  'F8 - P8'  'F8 - T7'  'F8 - T8'  'FC1 - C3'  'FC1 - C4'  'FC1 - CP5'  'FC1 - CP6'  'FC1 - FC2'  'FC1 - FC6'  'FC1 - O1'  'FC1 - O2'  'FC1 - Oz'  'FC1 - P3'  'FC1 - P4'  'FC1 - P7'  'FC1 - P8'  'FC1 - POz'  'FC1 - Pz'  'FC1 - T7'  'FC1 - T8'  'FC2 - C3'  'FC2 - C4'  'FC2 - CP1'  'FC2 - CP2'  'FC2 - CP5'  'FC2 - CP6'  'FC2 - Cz'  'FC2 - FC6'  'FC2 - O1'  'FC2 - O2'  'FC2 - Oz'  'FC2 - P3'  'FC2 - P4'  'FC2 - P7'  'FC2 - P8'  'FC2 - POz'  'FC2 - Pz'  'FC2 - T7'  'FC2 - T8'  'FC5 - C3'  'FC5 - C4'  'FC5 - CP1'  'FC5 - CP2'  'FC5 - CP5'  'FC5 - CP6'  'FC5 - Cz'  'FC5 - FC1'  'FC5 - FC6'  'FC5 - O1'  'FC5 - O2'  'FC5 - Oz'  'FC5 - P3'  'FC5 - P4'  'FC5 - P7'  'FC5 - P8'  'FC5 - POz'  'FC5 - Pz'  'FC5 - T7'  'FC5 - T8'  'FC6 - C3'  'FC6 - C4'  'FC6 - CP1'  'FC6 - CP2'  'FC6 - CP5'  'FC6 - CP6'  'FC6 - Cz'  'FC6 - O1'  'FC6 - P3'  'FC6 - P7'  'FC6 - P8'  'FC6 - POz'  'FC6 - T7'  'FC6 - T8'  'Fp1 - C3'  'Fp1 - C4'  'Fp1 - CP1'  'Fp1 - CP2'  'Fp1 - CP5'  'Fp1 - CP6'  'Fp1 - Cz'  'Fp1 - F3'  'Fp1 - F4'  'Fp1 - F7'  'Fp1 - F8'  'Fp1 - FC1'  'Fp1 - FC2'  'Fp1 - FC5'  'Fp1 - FC6'  'Fp1 - Fp2'  'Fp1 - Fpz'  'Fp1 - Fz'  'Fp1 - O1'  'Fp1 - O2'  'Fp1 - Oz'  'Fp1 - P3'  'Fp1 - P4'  'Fp1 - P7'  'Fp1 - P8'  'Fp1 - POz'  'Fp1 - Pz'  'Fp1 - T7'  'Fp1 - T8'  'Fp2 - C3'  'Fp2 - C4'  'Fp2 - CP1'  'Fp2 - CP2'  'Fp2 - CP5'  'Fp2 - CP6'  'Fp2 - Cz'  'Fp2 - F3'  'Fp2 - F4'  'Fp2 - F7'  'Fp2 - F8'  'Fp2 - FC1'  'Fp2 - FC2'  'Fp2 - FC5'  'Fp2 - FC6'  'Fp2 - Fz'  'Fp2 - O1'  'Fp2 - O2'  'Fp2 - Oz'  'Fp2 - P3'  'Fp2 - P4'  'Fp2 - P7'  'Fp2 - P8'  'Fp2 - POz'  'Fp2 - Pz'  'Fp2 - T7'  'Fp2 - T8'  'Fpz - C3'  'Fpz - C4'  'Fpz - CP1'  'Fpz - CP2'  'Fpz - CP5'  'Fpz - CP6'  'Fpz - Cz'  'Fpz - F3'  'Fpz - F4'  'Fpz - F7'  'Fpz - F8'  'Fpz - FC1'  'Fpz - FC2'  'Fpz - FC5'  'Fpz - FC6'  'Fpz - Fp2'  'Fpz - Fz'  'Fpz - O1'  'Fpz - O2'  'Fpz - Oz'  'Fpz - P3'  'Fpz - P4'  'Fpz - P7'  'Fpz - P8'  'Fpz - POz'  'Fpz - Pz'  'Fpz - T7'  'Fpz - T8'  'Fz - C3'  'Fz - C4'  'Fz - CP1'  'Fz - CP2'  'Fz - CP5'  'Fz - CP6'  'Fz - Cz'  'Fz - F4'  'Fz - F8'  'Fz - FC1'  'Fz - FC2'  'Fz - FC5'  'Fz - FC6'  'Fz - O1'  'Fz - O2'  'Fz - Oz'  'Fz - P3'  'Fz - P4'  'Fz - P7'  'Fz - P8'  'Fz - POz'  'Fz - Pz'  'Fz - T7'  'Fz - T8' |
| **Electrode Pairs Central ROI (7 electrodes)**  roi_central = {'Cz','C3','C4','CP1','CP2','CP5','CP6'} | | | |
| **Delta (157 pairs)** | **Theta (160 pairs)** | **Alpha (177 pairs)** | **Beta (157 pairs)** |
| 'C3 - C4'  'C3 - CP1'  'C3 - CP2'  'C3 - CP5'  'C3 - Cz'  'C3 - O2'  'C3 - Oz'  'C3 - P4'  'C3 - P8'  'C3 - Pz'  'C3 - T8'  'C4 - CP1'  'C4 - CP2'  'C4 - CP5'  'C4 - CP6'  'C4 - O1'  'C4 - O2'  'C4 - Oz'  'C4 - P3'  'C4 - P4'  'C4 - P7'  'C4 - P8'  'C4 - POz'  'C4 - Pz'  'C4 - T8'  'CP1 - CP2'  'CP1 - CP6'  'CP1 - O1'  'CP1 - O2'  'CP1 - Oz'  'CP1 - P3'  'CP1 - P4'  'CP1 - P7'  'CP1 - P8'  'CP1 - POz'  'CP1 - Pz'  'CP2 - CP6'  'CP2 - O2'  'CP2 - P3'  'CP2 - P4'  'CP2 - P7'  'CP2 - P8'  'CP2 - POz'  'CP2 - Pz'  'CP5 - CP1'  'CP5 - CP2'  'CP5 - CP6'  'CP5 - O1'  'CP5 - O2'  'CP5 - Oz'  'CP5 - P3'  'CP5 - P4'  'CP5 - P7'  'CP5 - P8'  'CP5 - POz'  'CP5 - Pz'  'CP6 - O1'  'CP6 - O2'  'CP6 - Oz'  'CP6 - P3'  'CP6 - P4'  'CP6 - P8'  'CP6 - POz'  'CP6 - Pz'  'Cz - C4'  'Cz - CP1'  'Cz - CP2'  'Cz - CP6'  'Cz - O1'  'Cz - O2'  'Cz - Oz'  'Cz - P3'  'Cz - P4'  'Cz - P7'  'Cz - P8'  'Cz - POz'  'Cz - Pz'  'Cz - T8'  'F3 - C3'  'F3 - C4'  'F3 - CP1'  'F3 - CP2'  'F3 - CP6'  'F3 - Cz'  'F4 - C3'  'F4 - C4'  'F4 - CP1'  'F4 - CP2'  'F4 - CP5'  'F4 - CP6'  'F4 - Cz'  'F7 - C3'  'F7 - C4'  'F7 - CP1'  'F7 - CP2'  'F7 - CP5'  'F7 - CP6'  'F7 - Cz'  'F8 - C3'  'F8 - CP1'  'F8 - CP5'  'F8 - Cz'  'FC1 - C3'  'FC1 - C4'  'FC1 - CP1'  'FC1 - CP2'  'FC1 - Cz'  'FC2 - C3'  'FC2 - C4'  'FC2 - CP1'  'FC2 - CP2'  'FC2 - CP5'  'FC2 - CP6'  'FC2 - Cz'  'FC5 - C3'  'FC5 - C4'  'FC5 - CP1'  'FC5 - CP2'  'FC5 - CP5'  'FC5 - Cz'  'FC6 - C3'  'FC6 - C4'  'FC6 - CP1'  'FC6 - CP2'  'FC6 - CP5'  'FC6 - Cz'  'Fp1 - C3'  'Fp1 - C4'  'Fp1 - CP1'  'Fp1 - CP2'  'Fp1 - CP6'  'Fp1 - Cz'  'Fp2 - C3'  'Fp2 - C4'  'Fp2 - CP1'  'Fp2 - CP2'  'Fp2 - CP6'  'Fp2 - Cz'  'Fpz - C3'  'Fpz - C4'  'Fpz - CP1'  'Fpz - CP2'  'Fpz - Cz'  'Fz - C4'  'Fz - CP1'  'Fz - CP2'  'Fz - CP6'  'Fz - Cz'  'T7 - C3'  'T7 - C4'  'T7 - CP1'  'T7 - CP2'  'T7 - CP6'  'T7 - Cz'  'T8 - CP1'  'T8 - CP2'  'T8 - CP5' | 'C3 - C4'  'C3 - CP2'  'C3 - CP5'  'C3 - CP6'  'C3 - Cz'  'C3 - O1'  'C3 - O2'  'C3 - Oz'  'C3 - P3'  'C3 - P4'  'C3 - P7'  'C3 - P8'  'C3 - POz'  'C3 - Pz'  'C3 - T8'  'C4 - CP2'  'C4 - CP5'  'C4 - CP6'  'C4 - O1'  'C4 - O2'  'C4 - Oz'  'C4 - P4'  'C4 - P7'  'C4 - P8'  'C4 - POz'  'C4 - Pz'  'C4 - T8'  'CP1 - CP2'  'CP1 - CP6'  'CP1 - O2'  'CP1 - P4'  'CP1 - P7'  'CP1 - P8'  'CP1 - Pz'  'CP2 - CP6'  'CP2 - O1'  'CP2 - O2'  'CP2 - Oz'  'CP2 - P3'  'CP2 - P4'  'CP2 - P7'  'CP2 - P8'  'CP2 - POz'  'CP2 - Pz'  'CP5 - CP1'  'CP5 - CP2'  'CP5 - CP6'  'CP5 - O1'  'CP5 - O2'  'CP5 - Oz'  'CP5 - P3'  'CP5 - P4'  'CP5 - P7'  'CP5 - P8'  'CP5 - POz'  'CP5 - Pz'  'CP6 - O1'  'CP6 - O2'  'CP6 - Oz'  'CP6 - P3'  'CP6 - P4'  'CP6 - P7'  'CP6 - P8'  'CP6 - POz'  'CP6 - Pz'  'Cz - C4'  'Cz - CP1'  'Cz - CP2'  'Cz - CP5'  'Cz - CP6'  'Cz - O1'  'Cz - O2'  'Cz - P3'  'Cz - P4'  'Cz - P7'  'Cz - P8'  'Cz - POz'  'Cz - Pz'  'Cz - T8'  'F3 - C4'  'F3 - CP2'  'F3 - CP5'  'F3 - CP6'  'F3 - Cz'  'F4 - C3'  'F4 - C4'  'F4 - CP1'  'F4 - CP2'  'F4 - CP5'  'F4 - CP6'  'F4 - Cz'  'F7 - C3'  'F7 - C4'  'F7 - CP1'  'F7 - CP2'  'F7 - CP5'  'F7 - CP6'  'F7 - Cz'  'F8 - C3'  'F8 - C4'  'F8 - CP1'  'F8 - CP2'  'F8 - CP5'  'F8 - CP6'  'F8 - Cz'  'FC1 - C3'  'FC1 - C4'  'FC1 - CP2'  'FC1 - CP5'  'FC1 - CP6'  'FC1 - Cz'  'FC2 - C3'  'FC2 - C4'  'FC2 - CP1'  'FC2 - CP2'  'FC2 - CP5'  'FC2 - CP6'  'FC2 - Cz'  'FC5 - C3'  'FC5 - C4'  'FC5 - CP1'  'FC5 - CP2'  'FC5 - CP5'  'FC5 - CP6'  'FC5 - Cz'  'FC6 - C3'  'FC6 - C4'  'FC6 - CP1'  'FC6 - CP2'  'FC6 - CP5'  'FC6 - CP6'  'FC6 - Cz'  'Fp1 - C4'  'Fp1 - CP2'  'Fp1 - CP6'  'Fp1 - Cz'  'Fp2 - C4'  'Fp2 - CP2'  'Fp2 - CP6'  'Fp2 - Cz'  'Fpz - C4'  'Fpz - CP2'  'Fpz - CP5'  'Fpz - CP6'  'Fpz - Cz'  'Fz - C4'  'Fz - CP2'  'Fz - CP5'  'Fz - CP6'  'Fz - Cz'  'T7 - C3'  'T7 - C4'  'T7 - CP1'  'T7 - CP2'  'T7 - CP5'  'T7 - CP6'  'T7 - Cz'  'T8 - CP1'  'T8 - CP2'  'T8 - CP6' | 'C3 - C4'  'C3 - CP1'  'C3 - CP2'  'C3 - CP5'  'C3 - CP6'  'C3 - Cz'  'C3 - O1'  'C3 - O2'  'C3 - Oz'  'C3 - P3'  'C3 - P4'  'C3 - P7'  'C3 - P8'  'C3 - POz'  'C3 - Pz'  'C3 - T8'  'C4 - CP1'  'C4 - CP2'  'C4 - CP5'  'C4 - CP6'  'C4 - O1'  'C4 - O2'  'C4 - Oz'  'C4 - P3'  'C4 - P4'  'C4 - P7'  'C4 - P8'  'C4 - POz'  'C4 - Pz'  'C4 - T8'  'CP1 - CP2'  'CP1 - CP6'  'CP1 - O1'  'CP1 - O2'  'CP1 - Oz'  'CP1 - P4'  'CP1 - P7'  'CP1 - P8'  'CP1 - POz'  'CP1 - Pz'  'CP2 - CP6'  'CP2 - O1'  'CP2 - O2'  'CP2 - Oz'  'CP2 - P3'  'CP2 - P4'  'CP2 - P7'  'CP2 - P8'  'CP2 - POz'  'CP2 - Pz'  'CP5 - CP1'  'CP5 - CP2'  'CP5 - CP6'  'CP5 - O1'  'CP5 - O2'  'CP5 - P3'  'CP5 - P4'  'CP5 - P7'  'CP5 - P8'  'CP5 - POz'  'CP5 - Pz'  'CP6 - O1'  'CP6 - O2'  'CP6 - Oz'  'CP6 - P3'  'CP6 - P4'  'CP6 - P7'  'CP6 - P8'  'CP6 - POz'  'CP6 - Pz'  'Cz - C4'  'Cz - CP1'  'Cz - CP2'  'Cz - CP5'  'Cz - CP6'  'Cz - O1'  'Cz - O2'  'Cz - Oz'  'Cz - P3'  'Cz - P4'  'Cz - P7'  'Cz - P8'  'Cz - POz'  'Cz - Pz'  'Cz - T8'  'F3 - C3'  'F3 - C4'  'F3 - CP1'  'F3 - CP2'  'F3 - CP5'  'F3 - CP6'  'F3 - Cz'  'F4 - C3'  'F4 - C4'  'F4 - CP1'  'F4 - CP2'  'F4 - CP5'  'F4 - CP6'  'F4 - Cz'  'F7 - C3'  'F7 - C4'  'F7 - CP1'  'F7 - CP2'  'F7 - CP5'  'F7 - CP6'  'F7 - Cz'  'F8 - C3'  'F8 - C4'  'F8 - CP1'  'F8 - CP2'  'F8 - CP5'  'F8 - Cz'  'FC1 - C3'  'FC1 - C4'  'FC1 - CP1'  'FC1 - CP2'  'FC1 - CP5'  'FC1 - CP6'  'FC1 - Cz'  'FC2 - C3'  'FC2 - C4'  'FC2 - CP1'  'FC2 - CP2'  'FC2 - CP5'  'FC2 - CP6'  'FC2 - Cz'  'FC5 - C4'  'FC5 - CP1'  'FC5 - CP2'  'FC5 - CP5'  'FC5 - CP6'  'FC5 - Cz'  'FC6 - C3'  'FC6 - C4'  'FC6 - CP1'  'FC6 - CP2'  'FC6 - CP5'  'FC6 - CP6'  'FC6 - Cz'  'Fp1 - C3'  'Fp1 - C4'  'Fp1 - CP1'  'Fp1 - CP2'  'Fp1 - CP5'  'Fp1 - CP6'  'Fp1 - Cz'  'Fp2 - C3'  'Fp2 - C4'  'Fp2 - CP1'  'Fp2 - CP2'  'Fp2 - CP5'  'Fp2 - CP6'  'Fp2 - Cz'  'Fpz - C3'  'Fpz - C4'  'Fpz - CP1'  'Fpz - CP2'  'Fpz - CP5'  'Fpz - CP6'  'Fpz - Cz'  'Fz - C3'  'Fz - C4'  'Fz - CP1'  'Fz - CP2'  'Fz - CP5'  'Fz - CP6'  'Fz - Cz'  'T7 - C3'  'T7 - C4'  'T7 - CP1'  'T7 - CP2'  'T7 - CP5'  'T7 - CP6'  'T7 - Cz'  'T8 - CP1'  'T8 - CP2'  'T8 - CP5' | 'C3 - C4'  'C3 - CP2'  'C3 - CP6'  'C3 - Cz'  'C3 - O1'  'C3 - O2'  'C3 - Oz'  'C3 - P3'  'C3 - P4'  'C3 - P7'  'C3 - P8'  'C3 - POz'  'C3 - Pz'  'C3 - T8'  'C4 - CP1'  'C4 - CP2'  'C4 - CP6'  'C4 - O1'  'C4 - O2'  'C4 - Oz'  'C4 - P4'  'C4 - P7'  'C4 - P8'  'C4 - POz'  'C4 - Pz'  'C4 - T8'  'CP1 - O1'  'CP1 - O2'  'CP1 - Oz'  'CP1 - P4'  'CP1 - Pz'  'CP2 - CP6'  'CP2 - O1'  'CP2 - O2'  'CP2 - Oz'  'CP2 - P4'  'CP2 - P8'  'CP2 - POz'  'CP2 - Pz'  'CP5 - CP6'  'CP5 - O1'  'CP5 - O2'  'CP5 - Oz'  'CP5 - P3'  'CP5 - P4'  'CP5 - P7'  'CP5 - P8'  'CP5 - POz'  'CP5 - Pz'  'CP6 - O2'  'CP6 - P4'  'CP6 - P7'  'CP6 - P8'  'CP6 - POz'  'CP6 - Pz'  'Cz - CP1'  'Cz - CP2'  'Cz - CP5'  'Cz - CP6'  'Cz - O1'  'Cz - O2'  'Cz - Oz'  'Cz - P3'  'Cz - P4'  'Cz - P7'  'Cz - P8'  'Cz - POz'  'Cz - Pz'  'Cz - T8'  'F3 - C3'  'F3 - C4'  'F3 - CP1'  'F3 - CP5'  'F3 - CP6'  'F3 - Cz'  'F4 - C3'  'F4 - CP1'  'F4 - CP2'  'F4 - CP5'  'F4 - CP6'  'F4 - Cz'  'F7 - C3'  'F7 - C4'  'F7 - CP1'  'F7 - CP2'  'F7 - CP5'  'F7 - CP6'  'F7 - Cz'  'F8 - C3'  'F8 - C4'  'F8 - CP1'  'F8 - CP2'  'F8 - CP5'  'F8 - CP6'  'F8 - Cz'  'FC1 - C3'  'FC1 - C4'  'FC1 - CP5'  'FC1 - CP6'  'FC2 - C3'  'FC2 - C4'  'FC2 - CP1'  'FC2 - CP2'  'FC2 - CP5'  'FC2 - CP6'  'FC2 - Cz'  'FC5 - C3'  'FC5 - C4'  'FC5 - CP1'  'FC5 - CP2'  'FC5 - CP5'  'FC5 - CP6'  'FC5 - Cz'  'FC6 - C3'  'FC6 - C4'  'FC6 - CP1'  'FC6 - CP2'  'FC6 - CP5'  'FC6 - CP6'  'FC6 - Cz'  'Fp1 - C3'  'Fp1 - C4'  'Fp1 - CP1'  'Fp1 - CP2'  'Fp1 - CP5'  'Fp1 - CP6'  'Fp1 - Cz'  'Fp2 - C3'  'Fp2 - C4'  'Fp2 - CP1'  'Fp2 - CP2'  'Fp2 - CP5'  'Fp2 - CP6'  'Fp2 - Cz'  'Fpz - C3'  'Fpz - C4'  'Fpz - CP1'  'Fpz - CP2'  'Fpz - CP5'  'Fpz - CP6'  'Fpz - Cz'  'Fz - C3'  'Fz - C4'  'Fz - CP1'  'Fz - CP2'  'Fz - CP5'  'Fz - CP6'  'Fz - Cz'  'T7 - C3'  'T7 - C4'  'T7 - CP1'  'T7 - CP5'  'T7 - CP6'  'T7 - Cz'  'T8 - CP2'  'T8 - CP5'  'T8 - CP6' |

**Supplementary Table S5. Model Fit Criteria for Connectivity Models (Predictor: Attachment Anxiety)**

| **Frequency Band** | **ROI** | **AIC** | **BIC** | **LogLikelihood** | **Deviance** |
| --- | --- | --- | --- | --- | --- |
| Delta | wPLI_frontal | -263.04 | -237.49 | 139.52 | -279.04 |
| Theta | wPLI_frontal | -341.05 | -315.50 | 178.52 | -357.05 |
| Alpha | wPLI_frontal | -247.28 | -221.74 | 131.64 | -263.28 |
| Beta | wPLI_frontal | -581.82 | -556.28 | 298.91 | -597.82 |
| Delta | wPLI_central | -392.42 | -366.88 | 204.21 | -408.42 |
| Theta | wPLI_central | -285.14 | -259.60 | 150.57 | -301.14 |
| Alpha | wPLI_central | -594.99 | -569.44 | 305.49 | -610.99 |
| Beta | wPLI_central | -316.74 | -291.20 | 166.37 | -332.74 |

ROI = Region of Interest; wPLI = Weighted Phase Lag Index. AIC = Akaike Information Criterion; BIC = Bayesian Information Criterion. LogLikelihood and Deviance correspond to model fit indices used to assess relative quality of linear mixed-effects models.

**Supplementary Table S6. Model Fit Criteria for Connectivity Models (Predictor: Attachment Avoidance)**

| **Frequency Band** | **ROI** | **AIC** | **BIC** | **LogLikelihood** | **Deviance** |
| --- | --- | --- | --- | --- | --- |
| Delta | wPLI_frontal | -264.23 | -238.68 | 140.11 | -280.23 |
| Theta | wPLI_frontal | -293.20 | -267.66 | 154.60 | -309.20 |
| Alpha | wPLI_frontal | -244.34 | -218.80 | 130.17 | -260.34 |
| Beta | wPLI_frontal | -632.19 | -606.64 | 324.09 | -648.19 |
| Delta | wPLI_central | -335.74 | -310.19 | 175.87 | -351.74 |
| Theta | wPLI_central | -283.60 | -258.06 | 149.80 | -299.60 |
| Alpha | wPLI_central | -647.20 | -621.66 | 331.60 | -663.20 |
| Beta | wPLI_central | -317.07 | -291.53 | 166.54 | -333.07 |

ROI = Region of Interest; wPLI = Weighted Phase Lag Index. AIC = Akaike Information Criterion; BIC = Bayesian Information Criterion. LogLikelihood and Deviance correspond to model fit indices used to assess relative quality of linear mixed-effects models.

**Supplementary Table S7. Descriptive Statistics for Self-Reported Arousal Across Conditions**

| **Condition** | **N** | **Min** | **Max** | **Range** | **Mean** | **SD** | **SE** | **95% CI** |
| --- | --- | --- | --- | --- | --- | --- | --- | --- |
| Natural Neutral | 60 | 1.00 | 4.73 | 3.73 | 2.093 | 0.978 | 0.126 | 1.84, 2.35 |
| Reappraise | 60 | 1.00 | 6.07 | 5.07 | 2.429 | 1.225 | 0.158 | 2.11, 2.75 |
| Suppress | 60 | 1.00 | 6.07 | 5.07 | 2.597 | 1.312 | 0.169 | 2.26, 2.94 |
| Natural Negative | 60 | 1.07 | 6.73 | 5.66 | 2.907 | 1.384 | 0.179 | 2.55, 3.27 |

*N* = number of participants. *Min* = minimum value; *Max* = maximum value; *Range* = difference between maximum and minimum values. *SD* = standard deviation; *SE* = standard error of the mean. *CI* = confidence interval (95%) for the mean.

**Supplementary Table S8. Shapiro-Wilk Test for Normality of Arousal Ratings by Condition**

| **Condition** | **W** | **p Value** | **Passed Normality (α = .05)?** | **p Value Summary** |
| --- | --- | --- | --- | --- |
| Natural Neutral | 0.8856 | <0.0001 | No | **** |
| Reappraise | 0.9027 | 0.0002 | No | *** |
| Suppress | 0.8824 | <0.0001 | No | **** |
| Natural Negative | 0.9199 | 0.0008 | No | *** |

*W* = Shapiro–Wilk test statistic. *p Value* = significance level of the test. “Passed Normality” indicates whether the data meet the assumption of normality at α = .05. Asterisks denote significance levels: ***p* < .001, ****p* < .0001.

**Supplementary Table S9. Post-Hoc Arousal Comparisons and Effect Sizes**

| **Comparison** | **Rank sum diff.** | **Summary** | **Adjusted p** | **Cohen’s d** |
| --- | --- | --- | --- | --- |
| Natural Neutral vs. Reappraise | -25.00 | ns | 0.4626 | 0.303 |
| Natural Neutral vs. Suppress | -48.50 | ** | 0.0036 | 0.436 |
| Natural Neutral vs. Natural Negative | -94.50 | **** | <0.0001 | 0.679 |
| Reappraise vs. Suppress | -23.50 | ns | 0.5794 | 0.132 |
| Reappraise vs. Natural Negative | -69.50 | **** | <0.0001 | 0.366 |
| Suppress vs. Natural Negative | -46.00 | ** | 0.0069 | 0.230 |

Pairwise post-hoc comparisons were conducted using Dunn’s test with Holm-adjusted *p*-values. Asterisks indicate the level of significance: **p* < .01, ****p* < .0001. *Cohen’s d* represents the standardized effect size for each pairwise difference in arousal ratings. “ns” = not significant.

**Supplementary Table S10. Suprathreshold Electrode Pairs in the Delta Band (1–3 Hz) per Emotion Regulation Condition**

| **Condition** | **n** | **Electrode Pair** | **ΔwPLI** |
| --- | --- | --- | --- |
| Reappraise | 1 | FC6 – FC2 | 0.0662 |
|  | 2 | P8 – T7 | 0.0739 |
|  | 3 | Oz – P7 | 0.0743 |
|  | 4 | Oz – POz | 0.0683 |
|  |  |  |  |
| Negative | 1 | F3 – Fp2 | 0.0913 |
|  | 2 | FC1 – Fp2 | 0.0769 |
|  | 3 | FC1 – F3 | 0.0756 |
|  | 4 | FC1 – FC5 | 0.0908 |
|  | 5 | CP1 – T8 | 0.0755 |
|  |  |  |  |
| Suppress | 1 | F7 – Fp1 | 0.0906 |
|  | 2 | CP1 – Fp1 | 0.0883 |
|  | 3 | Pz – Fp1 | 0.0692 |
|  | 4 | Fz – Fpz | 0.0718 |
|  | 5 | FC2 – F7 | 0.0797 |
|  | 6 | Pz – F3 | 0.0680 |
|  | 7 | Pz – Fz | 0.0781 |
|  | 8 | Oz – Fz | 0.0652 |
|  | 9 | CP1 – F4 | 0.0786 |
|  | 10 | Pz – F4 | 0.0788 |
|  | 11 | POz – F4 | 0.0760 |
|  | 12 | Oz – F4 | 0.0838 |
|  | 13 | CP1 – F8 | 0.0785 |
|  | 14 | FC2 – FC5 | 0.0656 |
|  | 15 | Pz – FC5 | 0.0643 |
|  | 16 | FC6 – FC2 | 0.0772 |
|  | 17 | T7 – FC2 | 0.0735 |
|  | 18 | C3 – FC2 | 0.0666 |
|  | 19 | CP1 – FC2 | 0.0838 |
|  | 20 | P7 – FC2 | 0.1084 |
|  | 21 | Pz – FC2 | 0.0835 |
|  | 22 | POz – FC2 | 0.0769 |
|  | 23 | Oz – FC2 | 0.0725 |
|  | 24 | O2 – FC2 | 0.0700 |
|  | 25 | Cz – FC6 | 0.0855 |
|  | 26 | CP1 – FC6 | 0.0674 |
|  | 27 | Pz – T7 | 0.0689 |
|  | 28 | POz – P7 | 0.0826 |

Electrode pairs showing increased functional connectivity (ΔwPLI) relative to the Neutral condition, exceeding the data-driven threshold (95th percentile) for the delta band.

**Supplementary Table S11. Suprathreshold Electrode Pairs in the Theta Band (4–8 Hz) – Reappraise Condition Only**

| **Condition** | **n** | **Electrode Pair** | **ΔwPLI** |
| --- | --- | --- | --- |
| Reappraise | 1 | FC2 – F7 | 0.0609 |
|  | 2 | P8 – F7 | 0.0598 |
|  | 3 | FC6 – Fz | 0.0604 |
|  | 4 | P4 – Fz | 0.0602 |
|  | 5 | FC2 – F4 | 0.0591 |
|  | 6 | FC2 – F8 | 0.0718 |
|  | 7 | FC6 – F8 | 0.0994 |
|  | 8 | P4 – FC5 | 0.0696 |
|  | 9 | O2 – FC5 | 0.0585 |
|  | 10 | T8 – FC1 | 0.0582 |
|  | 11 | P4 – FC1 | 0.0730 |
|  | 12 | P8 – FC1 | 0.0734 |
|  | 13 | FC6 – FC2 | 0.0640 |
|  | 14 | Pz – FC2 | 0.0617 |
|  | 15 | P4 – FC2 | 0.0641 |
|  | 16 | P8 – FC2 | 0.0703 |
|  | 17 | POz – FC2 | 0.0580 |
|  | 18 | Cz – T7 | 0.0663 |
|  | 19 | P4 – T7 | 0.0583 |
|  | 20 | P8 – T7 | 0.0663 |
|  | 21 | P8 – C3 | 0.0778 |
|  | 22 | P4 – Cz | 0.0584 |
|  | 23 | P8 – Cz | 0.0860 |
|  | 24 | CP6 – C4 | 0.0595 |
|  | 25 | P8 – C4 | 0.0703 |
|  | 26 | P8 – CP2 | 0.0652 |
|  | 27 | O2 – CP2 | 0.0698 |
|  | 28 | P8 – CP6 | 0.0804 |
|  | 29 | O1 – CP6 | 0.0632 |
|  | 30 | Oz – CP6 | 0.0587 |
|  | 31 | O2 – CP6 | 0.0664 |
|  | 32 | POz – Pz | 0.0587 |
|  | 33 | O1 – P4 | 0.0616 |
|  | 34 | Oz – P4 | 0.0650 |
|  | 35 | O2 – P4 | 0.0863 |

Electrode pairs showing increased functional connectivity (ΔwPLI) relative to the Neutral condition, exceeding the data-driven threshold (0.0573) for the theta band. No suprathreshold pairs were observed for the Negative or Suppress conditions.

**Supplementary Table S12. Suprathreshold Electrode Pairs in the Alpha Band (9–12 Hz)**

| **Condition** | **n** | **Electrode Pair** | **ΔwPLI** |
| --- | --- | --- | --- |
| Reappraise | 1 | FC2 – F7 | 0.0891 |
|  | 2 | Fz – F3 | 0.0841 |
|  | 3 | FC2 – FC5 | 0.0852 |
|  | 4 | T7 – FC2 | 0.0794 |
|  | 5 | C4 – Cz | 0.0780 |
|  | 6 | CP6 – CP5 | 0.0844 |
|  | 7 | Oz – CP6 | 0.0845 |
|  | 8 | O2 – CP6 | 0.0743 |
|  | 9 | O1 – P3 | 0.0775 |
|  | 10 | O1 – P8 | 0.0941 |
|  | 11 | Oz – P8 | 0.0853 |
|  | 12 | O2 – P8 | 0.0802 |
|  |  |  |  |
| Negative | 1 | FC2 – Fpz | 0.0791 |
|  | 2 | Cz – F3 | 0.0958 |
|  | 3 | Cz – Fz | 0.0795 |
|  | 4 | C4 – FC6 | 0.0860 |
|  |  |  |  |
| Suppress | 1 | F3 – Fp1 | 0.0944 |
|  | 2 | Fz – Fp1 | 0.0920 |
|  | 3 | FC1 – Fp1 | 0.0751 |
|  | 4 | T7 – Fp1 | 0.0827 |
|  | 5 | FC5 – Fp2 | 0.0820 |
|  | 6 | FC6 – Fp2 | 0.0822 |
|  | 7 | T7 – Fp2 | 0.0984 |
|  | 8 | CP2 – Fp2 | 0.0895 |
|  | 9 | Cz – F7 | 0.0745 |
|  | 10 | Fz – F3 | 0.0924 |
|  | 11 | FC5 – F3 | 0.0764 |
|  | 12 | FC2 – F3 | 0.0827 |
|  | 13 | Cz – F3 | 0.0925 |
|  | 14 | CP5 – F3 | 0.0839 |
|  | 15 | FC5 – F4 | 0.0785 |
|  | 16 | Cz – F4 | 0.0788 |
|  | 17 | Cz – F8 | 0.0842 |
|  | 18 | FC2 – FC5 | 0.0807 |
|  | 19 | C3 – FC1 | 0.0747 |
|  | 20 | Cz – FC1 | 0.0842 |
|  | 21 | C4 – FC1 | 0.0814 |
|  | 22 | T7 – FC2 | 0.0922 |
|  | 23 | Cz – FC6 | 0.0749 |
|  | 24 | C4 – FC6 | 0.0825 |
|  | 25 | T8 – FC6 | 0.0799 |
|  | 26 | CP2 – FC6 | 0.0941 |
|  | 27 | Pz – C3 | 0.0786 |
|  | 29 | P4 – CP2 | 0.0824 |

Electrode pairs showing increased functional connectivity (ΔwPLI) relative to the Neutral condition, exceeding the data-driven threshold (0.0741) for the alpha band.

**Supplementary Table S13. Suprathreshold Electrode Pairs in the Beta Band (15–30 Hz)**

| **Condition** | **n** | **Electrode Pair** | **ΔwPLI** |
| --- | --- | --- | --- |
| Reappraise | 1 | F3 – Fp1 | 0.0382 |
|  | 2 | T8 – Fp1 | 0.0314 |
|  | 3 | CP6 – Fp1 | 0.0318 |
|  | 4 | POz – Fp1 | 0.0318 |
|  | 5 | F4 – Fpz | 0.0365 |
|  | 6 | T8 – Fpz | 0.0368 |
|  | 7 | CP6 – Fpz | 0.0487 |
|  | 8 | F4 – Fp2 | 0.0312 |
|  | 9 | CP6 – Fp2 | 0.0356 |
|  | 10 | T8 – F7 | 0.0314 |
|  | 11 | POz – F7 | 0.0318 |
|  | 12 | F4 – F3 | 0.0361 |
|  | 13 | F8 – F3 | 0.0445 |
|  | 14 | FC6 – F3 | 0.0447 |
|  | 15 | T8 – F3 | 0.0425 |
|  | 16 | P7 – F3 | 0.0333 |
|  | 17 | P3 – F3 | 0.0374 |
|  | 18 | F4 – Fz | 0.0415 |
|  | 19 | P7 – Fz | 0.0416 |
|  | 20 | P3 – Fz | 0.0332 |
|  |  |  | 0.0312 |
|  |  | F3 – F4 | 0.0361 |
|  | 21 | FC1 – F4 | 0.0355 |
|  | 22 | P7 – F4 | 0.0331 |
|  | 23 | Cz – F8 | 0.0314 |
|  | 24 | P7 – FC5 | 0.0334 |
|  | 25 | P3 – FC5 | 0.0366 |
|  | 26 | FC6 – FC1 | 0.0399 |
|  | 27 | P8 – FC1 | 0.0344 |
|  | 28 | CP6 – Cz | 0.0335 |
|  | 29 | P7 – Cz | 0.0369 |
|  |  |  |  |
| Suppress | 1 | T7 – Fp1 | 0.0338 |
|  | 2 | P8 – F4 | 0.0316 |
|  | 3 | P8 – FC2 | 0.0411 |
|  | 4 | P8 – T7 | 0.0319 |
|  | 5 | P8 – Cz | 0.0361 |
|  | 6 | CP6 – C4 | 0.0391 |
|  | 7 | P7 – CP5 | 0.0331 |
|  | 8 | O1 – CP5 | 0.0448 |
|  | 9 | P8 – CP2 | 0.0329 |
|  | 10 | O2 – CP2 | 0.0314 |

Electrode pairs showing increased functional connectivity (ΔwPLI) relative to the Neutral condition, exceeding the data-driven threshold (0.0312) for the beta band. No suprathreshold pairs were observed for the Negative or Suppress conditions.

**Supplementary Table S14. Fixed effects for Frontal wPLI ROI across frequency bands (predictor: attachment anxiety)**

| **Frequency Band: Delta (1-3 Hz)** | | | | | | | |
| --- | --- | --- | --- | --- | --- | --- | --- |
| **Name** | **Estimate** | **SE** | **tStat** | **DF** | **pValue** | **FDR** | **95% CI** |
| (Intercept) | -0.0303 | 0.0413 | -0.7345 | 174 | 0.4636 | 0.8747 | [-0.11, 0.05] |
| Condition: Reappraise | 0.0208 | 0.0347 | 0.5990 | 174 | 0.5500 | 0.9087 | [-0.05, 0.09] |
| Condition: Suppress | 0.0426 | 0.0347 | 1.2257 | 174 | 0.2220 | 0.6107 | [-0.03, 0.11] |
| Attachment Anxiety | 0.0078 | 0.0098 | 0.7909 | 174 | 0.4301 | 0.8393 | [-0.01, 0.03] |
| Condition: Reapp*Att Anxiety | -0.0039 | 0.0083 | -0.4704 | 174 | 0.6386 | 0.9372 | [-0.02, 0.01] |
| Condition: Supp*Att Anxiety | -0.0030 | 0.0083 | -0.3623 | 174 | 0.7175 | 0.9584 | [-0.02, 0.01] |
| **Frequency Band: Theta (4-8 Hz)** | | | | | | | |
| **Name** | **Estimate** | **SE** | **tStat** | **DF** | **pValue** | **FDR** | **95% CI** |
| **(Intercept)** | **0.0910** | **0.0288** | **3.1592** | **174** | **0.0019** | **0.0223** | **[0.03, 0.15]** |
| **Condition: Reappraise** | **0.1393** | **0.0321** | **4.3356** | **174** | **<0.0001** | **0.0017** | **[0.08, 0.20]** |
| Condition: Suppress | 0.0276 | 0.0321 | 0.8605 | 174 | 0.3907 | 0.7837 | [-0.04, 0.09] |
| **Attachment Anxiety** | **-0.0247** | **0.0069** | **-3.5988** | **174** | **0.0004** | **0.0072** | **[-0.04, -0.01]** |
| **Condition: Reapp*Att Anxiety** | **-0.0291** | **0.0076** | **-3.8077** | **174** | **0.0002** | **0.0047** | **[-0.04, -0.01]** |
| Condition: Supp*Att Anxiety | -0.0105 | 0.0076 | -1.3744 | 174 | 0.1711 | 0.5552 | [-0.03, 0.00] |
| **Frequency Band: Alpha (9-12 Hz)** | | | | | | | |
| **Name** | **Estimate** | **SE** | **tStat** | **DF** | **pValue** | **FDR** | **95% CI** |
| (Intercept) | 0.0569 | 0.0407 | 1.3998 | 174 | 0.1634 | 0.5684 | [-0.02, 0.14] |
| Condition: Reappraise | 0.0218 | 0.0381 | 0.5715 | 174 | 0.5684 | 0.9300 | [-0.05, 0.10] |
| Condition: Suppress | 0.0516 | 0.0381 | 1.3546 | 174 | 0.1773 | 0.5747 | [-0.02, 0.13] |
| Attachment Anxiety | -0.0117 | 0.0097 | -1.2128 | 174 | 0.2269 | 0.6107 | [-0.03, 0.01] |
| Condition: Reapp*Att Anxiety | -0.0083 | 0.0091 | -0.9135 | 174 | 0.3623 | 0.7800 | [-0.03, 0.01] |
| Condition: Supp*Att Anxiety | -0.0054 | 0.0091 | -0.5973 | 174 | 0.5511 | 0.9144 | [-0.02, 0.01] |
| **Frequency Band: Beta (15-30 Hz)** | | | | | | | |
| **Name** | **Estimate** | **SE** | **tStat** | **DF** | **pValue** | **FDR** | **95% CI** |
| (Intercept) | 0.0101 | 0.0157 | 0.6424 | 174 | 0.5215 | 0.8997 | [-0.02, 0.04] |
| Condition: Reappraise | 0.0165 | 0.0154 | 1.0726 | 174 | 0.2849 | 0.6726 | [-0.01, 0.05] |
| Condition: Suppress | 0.0070 | 0.0154 | 0.4528 | 174 | 0.6512 | 0.9442 | [-0.02, 0.04] |
| Attachment Anxiety | -0.0032 | 0.0037 | -0.8475 | 174 | 0.3979 | 0.7837 | [-0.01, 0.00] |
| Condition: Reapp*Att Anxiety | 0.0003 | 0.0037 | 0.0867 | 174 | 0.9310 | 0.9831 | [-0.01, 0.01] |
| Condition: Supp*Att Anxiety | 0.0008 | 0.0037 | 0.2075 | 174 | 0.8358 | 0.9702 | [-0.01, 0.01] |

Bolded coefficients indicate statistically significant effects or interactions (p < .05). SE = standard error. CI = confidence interval (95%). FDR = false discovery rate, corrected using the Benjamini-Hochberg procedure. Att = Attachment.

**Supplementary Table S15. Fixed effects for Central wPLI ROI across frequency bands (predictor: attachment anxiety)**

| **Frequency Band: Delta (1-3 Hz)** | | | | | | | |
| --- | --- | --- | --- | --- | --- | --- | --- |
| **Name** | **Estimate** | **SE** | **tStat** | **DF** | **pValue** | **FDR** | **95% CI** |
| (Intercept) | 0.0127 | 0.0348 | 0.3647 | 174 | 0.7158 | 0.9606 | [-0.06, 0.08] |
| Condition: Reappraise | -0.0009 | 0.0304 | -0.0312 | 174 | 0.9752 | 0.9916 | [-0.06, 0.06] |
| Condition: Suppress | 0.0281 | 0.0304 | 0.9244 | 174 | 0.3566 | 0.7655 | [-0.03, 0.09] |
| Attachment Anxiety | -0.0016 | 0.0083 | -0.1884 | 174 | 0.8508 | 0.9748 | [-0.02, 0.01] |
| Condition: Reapp*Att Anxiety | -0.0015 | 0.0072 | -0.2051 | 174 | 0.8378 | 0.9723 | [-0.02, 0.01] |
| Condition: Supp*Att Anxiety | -0.0031 | 0.0072 | -0.4311 | 174 | 0.6670 | 0.9274 | [-0.02, 0.01] |
| **Frequency Band: Theta (4-8 Hz)** | | | | | | | |
| **Name** | **Estimate** | **SE** | **tStat** | **DF** | **pValue** | **FDR** | **95% CI** |
| **(Intercept)** | **0.0962** | **0.0244** | **3.9449** | **174** | **<0.0001** | **0.0011** | **[0.05, 0.14]** |
| **Condition: Reappraise** | **0.1187** | **0.0288** | **4.1143** | **174** | **<0.0001** | **0.0011** | **[0.06, 0.18]** |
| Condition: Suppress | 0.0147 | 0.0288 | 0.5100 | 174 | 0.6107 | 0.9204 | [-0.04, 0.07] |
| **Attachment Anxiety** | **-0.0238** | **0.0058** | **-4.0944** | **174** | **<0.0001** | **0.0011** | **[-0.04, -0.01]** |
| **Condition: Reapp*Att Anxiety** | **-0.0257** | **0.0069** | **-3.7377** | **174** | **0.0003** | **0.0043** | **[-0.04, -0.01]** |
| Condition: Supp*Att Anxiety. | -0.0079 | 0.0069 | -1.1561 | 174 | 0.2492 | 0.6244 | [-0.02, 0.01] |
| **Frequency Band: Alpha (9-12 Hz)** | | | | | | | |
| **Name** | **Estimate** | **SE** | **tStat** | **DF** | **pValue** | **FDR** | **95% CI** |
| (Intercept) | 0.0389 | 0.0359 | 1.0822 | 174 | 0.2806 | 0.6606 | [-0.03, 0.11] |
| Condition: Reappraise | 0.0213 | 0.0349 | 0.6105 | 174 | 0.5423 | 0.9052 | [-0.05, 0.09] |
| Condition: Suppress | 0.0597 | 0.0349 | 1.7104 | 174 | 0.0890 | 0.3451 | [-0.01, 0.13] |
| Attachment Anxiety | -0.0063 | 0.0086 | -0.7306 | 174 | 0.4660 | 0.8722 | [-0.02, 0.01] |
| Condition: Reapp* Att Anxiety | -0.0047 | 0.0083 | -0.5677 | 174 | 0.5710 | 0.9123 | [-0.02, 0.01] |
| Condition: Supp*Att Anxiety | -0.0096 | 0.0083 | -1.1548 | 174 | 0.2498 | 0.6244 | [-0.03, 0.01] |
| **Frequency Band: Beta (15-30 Hz)** | | | | | | | |
| **Name** | **Estimate** | **SE** | **tStat** | **DF** | **pValue** | **FDR** | **95% CI** |
| (Intercept) | 0.0018 | 0.0151 | 0.1196 | 174 | 0.9050 | 0.9881 | [-0.03, 0.03] |
| Condition: Reappraise | 0.0102 | 0.0149 | 0.6891 | 174 | 0.4917 | 0.8882 | [-0.02, 0.04] |
| Condition: Suppress | 0.0125 | 0.0149 | 0.8395 | 174 | 0.4024 | 0.7995 | [-0.02, 0.04] |
| Attachment Att Anxiety | -0.0022 | 0.0036 | -0.6132 | 174 | 0.5406 | 0.9031 | [-0.01, 0.00] |
| Condition: Reapp*Att Anxiety | 0.0019 | 0.0035 | 0.5479 | 174 | 0.5845 | 0.9181 | [-0.01, 0.01] |
| Condition: Supp*Att Anxiety | 0.0010 | 0.0035 | 0.2798 | 174 | 0.7800 | 0.9803 | [-0.01, 0.01] |

Bolded coefficients indicate statistically significant effects or interactions (p < .05). SE = standard error. CI = confidence interval (95%). FDR = false discovery rate, corrected using the Benjamini-Hochberg procedure. Att = Attachment.

**Supplementary Table S16. Fixed effects for Frontal wPLI ROI across frequency bands (predictor: attachment avoidance)**

| **Frequency Band: Delta (1–3 Hz)** | | | | | | | |
| --- | --- | --- | --- | --- | --- | --- | --- |
| **Name** | **Estimate** | **SE** | **tStat** | **DF** | **pValue** | **FDR** | **95% CI** |
| (Intercept) | 0.0272 | 0.0461 | 0.5913 | 174 | 0.5551 | 0.9604 | [-0.06, 0.12] |
| Condition: Reappraise | 0.0169 | 0.0389 | 0.4337 | 174 | 0.6651 | 0.9851 | [-0.06, 0.09] |
| Condition: Suppress | 0.0595 | 0.0389 | 1.5291 | 174 | 0.1281 | 0.7802 | [-0.02, 0.14] |
| Attachment Avoidance | -0.0102 | 0.0155 | -0.6595 | 174 | 0.5105 | 0.9583 | [-0.04, 0.02] |
| Condition: Reapp*Att Avoidance | -0.0039 | 0.0131 | -0.3011 | 174 | 0.7637 | 0.9928 | [-0.03, 0.02] |
| Condition: Supp*Att Avoidance | -0.0103 | 0.0131 | -0.7865 | 174 | 0.4326 | 0.9417 | [-0.04, 0.02] |
| **Frequency Band: Theta (4–8 Hz)** | | | | | | | |
| **Name** | **Estimate** | **SE** | **tStat** | **DF** | **pValue** | **FDR** | **95% CI** |
| (Intercept) | -0.0268 | 0.0398 | -0.6738 | 174 | 0.5013 | 0.9565 | [-0.11, 0.05] |
| Condition: Reappraise | 0.0107 | 0.0379 | 0.2825 | 174 | 0.7779 | 0.9933 | [-0.06, 0.09] |
| Condition: Suppress | -0.0563 | 0.0379 | -1.4836 | 174 | 0.1397 | 0.8009 | [-0.13, 0.02] |
| Attachment Avoidance | 0.0088 | 0.0134 | 0.6575 | 174 | 0.5117 | 0.9583 | [-0.02, 0.03] |
| Condition: Reapp*Att Avoidance | 0.0066 | 0.0128 | 0.5153 | 174 | 0.6070 | 0.9733 | [-0.02, 0.03] |
| Condition: Supp*Att Avoidance | 0.0161 | 0.0128 | 1.2566 | 174 | 0.2106 | 0.8689 | [-0.01, 0.04] |
| **Frequency Band: Alpha (9–12 Hz)** | | | | | | | |
| **Name** | **Estimate** | **SE** | **tStat** | **DF** | **pValue** | **FDR** | **95% CI** |
| (Intercept) | -0.0017 | 0.0465 | -0.0372 | 174 | 0.9704 | 0.9984 | [-0.09, 0.09] |
| Condition: Reappraise | 0.0029 | 0.0426 | 0.0681 | 174 | 0.9458 | 0.9976 | [-0.08, 0.09] |
| Condition: Suppress | -0.0027 | 0.0426 | -0.0640 | 174 | 0.9491 | 0.9976 | [-0.09, 0.08] |
| Attachment Avoidance | 0.0051 | 0.0157 | 0.3280 | 174 | 0.7433 | 0.9832 | [-0.03, 0.04] |
| Condition: Reapp*Att Avoidance | -0.0046 | 0.0144 | -0.3191 | 174 | 0.7501 | 0.9843 | [-0.03, 0.02] |
| Condition: Supp*Att Avoidance | 0.0123 | 0.0144 | 0.8579 | 174 | 0.3921 | 0.9033 | [-0.02, 0.04] |
| **Frequency Band: Beta (15–30 Hz)** | | | | | | | |
| **Name** | **Estimate** | **SE** | **tStat** | **DF** | **pValue** | **FDR** | **95% CI** |
| (Intercept) | -0.0377 | 0.0149 | -2.5263 | 174 | 0.0124 | 0.2428 | [-0.07, -0.01] |
| Condition: Reappraise | 0.0173 | 0.0154 | 1.1275 | 174 | 0.2611 | 0.7912 | [-0.01, 0.05] |
| **Condition: Suppress** | **-0.0594** | **0.0154** | **-3.8648** | **174** | **<0.0001** | **0.0069** | **[-0.09, -0.03]** |
| Attachment Avoidance | 0.0130 | 0.0050 | 2.5943 | 174 | 0.0103 | 0.2306 | [0.00, 0.02] |
| Condition: Reapp*Att Avoidance | 0.0001 | 0.0052 | 0.0277 | 174 | 0.9779 | 0.9989 | [-0.01, 0.01] |
| **Condition: Supp*Att Avoidance** | **0.0253** | **0.0052** | **4.8773** | **174** | **<0.0001** | **0.0001** | **[0.02, 0.04]** |

Bolded coefficients indicate statistically significant effects or interactions (p < .05). SE = standard error. CI = confidence interval (95%). FDR = false discovery rate, corrected using the Benjamini-Hochberg procedure. Att = Attachment.

**Supplementary Table S17. Fixed effects for Central wPLI ROI across frequency bands (predictor: attachment avoidance)**

| **Frequency Band: Delta (1–3 Hz)** | | | | | | | |
| --- | --- | --- | --- | --- | --- | --- | --- |
| **Name** | **Estimate** | **SE** | **tStat** | **DF** | **pValue** | **FDR** | **95% CI** |
| (Intercept) | 0.0279 | 0.0390 | 0.7164 | 174 | 0.4747 | 0.9315 | [-0.05, 0.10] |
| Condition: Reappraise | -0.0128 | 0.0341 | -0.3753 | 174 | 0.7079 | 0.9815 | [-0.08, 0.05] |
| Condition: Suppress | 0.0246 | 0.0341 | 0.7202 | 174 | 0.4724 | 0.9315 | [-0.04, 0.09] |
| Attachment Avoidance | -0.0077 | 0.0131 | -0.5873 | 174 | 0.5578 | 0.9512 | [-0.03, 0.02] |
| Condition: Reapp*Att Avoidance | 0.0023 | 0.0115 | 0.1973 | 174 | 0.8438 | 0.9892 | [-0.02, 0.02] |
| Condition: Supp*Att Avoidance | -0.0030 | 0.0115 | -0.2631 | 174 | 0.7928 | 0.9853 | [-0.03, 0.02] |
| **Frequency Band: Theta (4–8 Hz)** | | | | | | | |
| **Name** | **Estimate** | **SE** | **tStat** | **DF** | **pValue** | **FDR** | **95% CI** |
| (Intercept) | -0.0213 | 0.0349 | -0.6112 | 174 | 0.5418 | 0.9456 | [-0.09, 0.05] |
| Condition: Reappraise | 0.0250 | 0.0341 | 0.7339 | 174 | 0.4640 | 0.9201 | [-0.04, 0.09] |
| Condition: Suppress | -0.0398 | 0.0341 | -1.1668 | 174 | 0.2449 | 0.7608 | [-0.11, 0.03] |
| Attachment Avoidance | 0.0100 | 0.0118 | 0.8480 | 174 | 0.3976 | 0.7238 | [-0.01, 0.03] |
| Condition: Reapp*Att Avoidance | -0.0014 | 0.0115 | -0.1193 | 174 | 0.9052 | 0.9937 | [-0.02, 0.02] |
| Condition: Supp*Att Avoidance | 0.0089 | 0.0115 | 0.7741 | 174 | 0.4400 | 0.8943 | [-0.01, 0.03] |
| **Frequency Band: Alpha (9–12 Hz)** | | | | | | | |
| **Name** | **Estimate** | **SE** | **tStat** | **DF** | **pValue** | **FDR** | **95% CI** |
| (Intercept) | -0.0016 | 0.0407 | -0.0405 | 174 | 0.9678 | 0.9987 | [-0.08, 0.08] |
| Condition: Reappraise | 0.0214 | 0.0391 | 0.5474 | 174 | 0.5848 | 0.9572 | [-0.06, 0.10] |
| Condition: Suppress | -0.0060 | 0.0391 | -0.1525 | 174 | 0.8790 | 0.9945 | [-0.08, 0.07] |
| Attachment Avoidance | 0.0061 | 0.0137 | 0.4471 | 174 | 0.6554 | 0.9732 | [-0.02, 0.03] |
| Condition: Reapp*Att Avoidance | -0.0066 | 0.0132 | -0.4980 | 174 | 0.6191 | 0.9644 | [-0.03, 0.02] |
| Condition: Supp*Att Avoidance | 0.0107 | 0.0132 | 0.8102 | 174 | 0.4190 | 0.8679 | [-0.02, 0.04] |
| **Frequency Band: Beta (15–30 Hz)** | | | | | | | |
| **Name** | **Estimate** | **SE** | **tStat** | **DF** | **pValue** | **FDR** | **95% CI** |
| (Intercept) | -0.0442 | 0.0144 | -3.0763 | 174 | 0.0024 | 0.0617 | [-0.07, -0.02] |
| Condition: Reappraise | 0.0280 | 0.0147 | 1.9031 | 174 | 0.0587 | 0.2956 | [-0.00, 0.06] |
| **Condition: Suppress** | **-0.0474** | **0.0147** | **-3.2241** | **174** | **0.0015** | **0.0413** | **[-0.08, -0.02]** |
| Attachment Avoidance | 0.0137 | 0.0048 | 2.8358 | 174 | 0.0051 | 0.1315 | [0.00, 0.02] |
| Condition: Reapp*Att Avoidance | -0.0038 | 0.0050 | -0.7635 | 174 | 0.4462 | 0.8881 | [-0.01, 0.01] |
| **Condition: Supp*Att Avoidance** | **0.0232** | **0.0050** | **4.6852** | **174** | **<0.0001** | **0.0010** | **[0.01, 0.03]** |

Bolded coefficients indicate statistically significant effects or interactions (p < .05). SE = standard error. CI = confidence interval (95%). FDR = false discovery rate, corrected using the Benjamini-Hochberg procedure. Att = Attachment.

**Supplementary Figures**

**
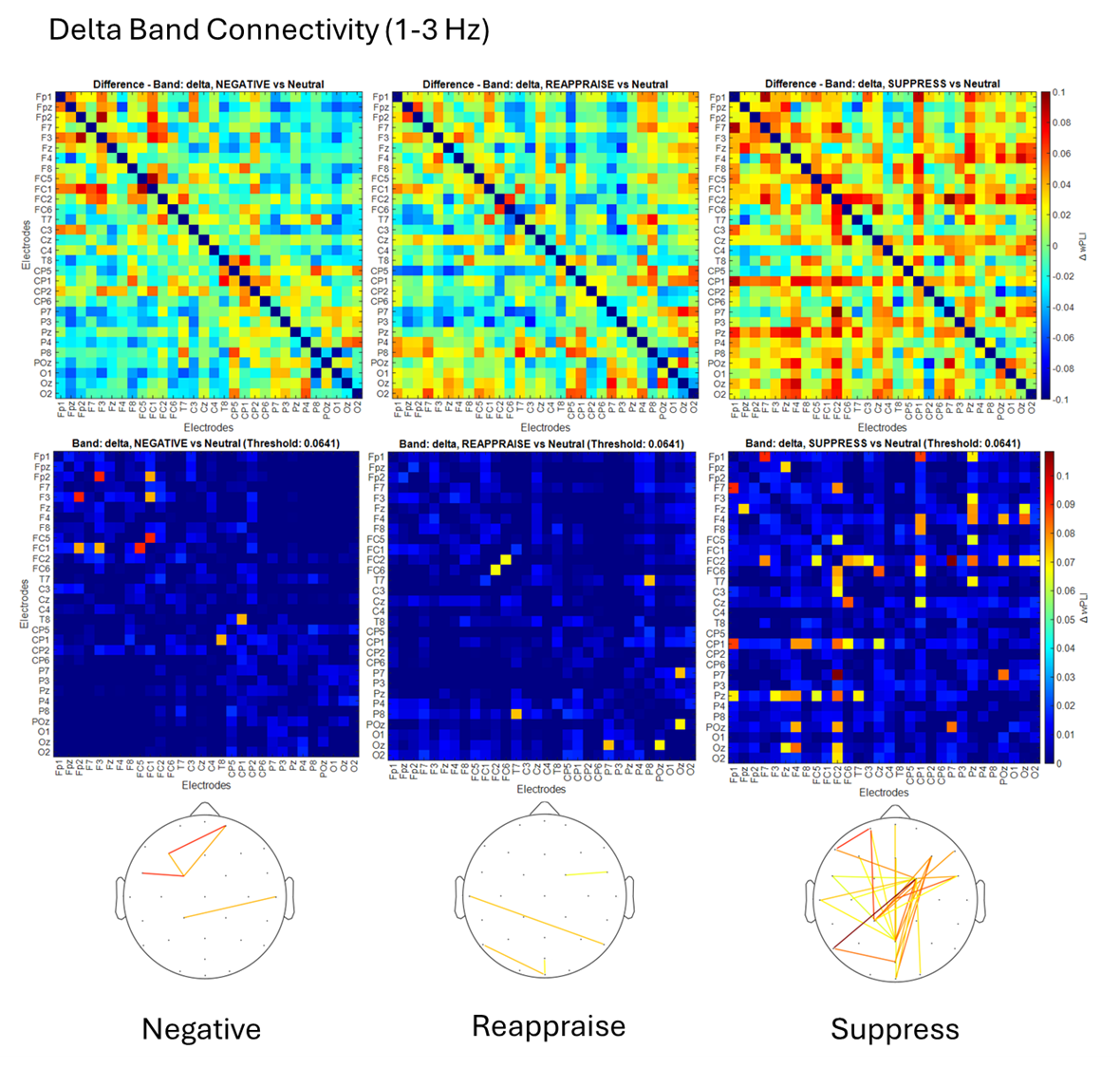
**

**Figure S1. Delta Band Connectivity (1–3 Hz) During Emotion Regulation Conditions.** *Top row*: Group-level difference matrices showing changes in debiased weighted Phase Lag Index (wPLI) connectivity in the delta band for each emotion regulation condition (Negative, Reappraise, Suppress) relative to the Neutral baseline. Warmer colors indicate stronger increases in phase synchrony between electrode pairs (ΔwPLI). Colorbar range: –0.1 to 0.1. *Middle row*: Thresholded difference matrices based on the 95th percentile of all positive differences across participants and conditions. Only electrode pairs with ΔwPLI exceeding the data-driven threshold (0.0641) are shown, with values below this cutoff attenuated or masked. Colorbar range: 0 to 0.09. *Bottom row*: Scalp maps depicting the topographical distribution of suprathreshold functional connections in each condition. Colored lines represent electrode pairs showing increased delta-band connectivity relative to Neutral, with warmer hues reflecting higher ΔwPLI values. These results highlight a pattern of widespread delta synchrony during suppression, with more restricted connectivity observed under negative viewing and reappraisal.


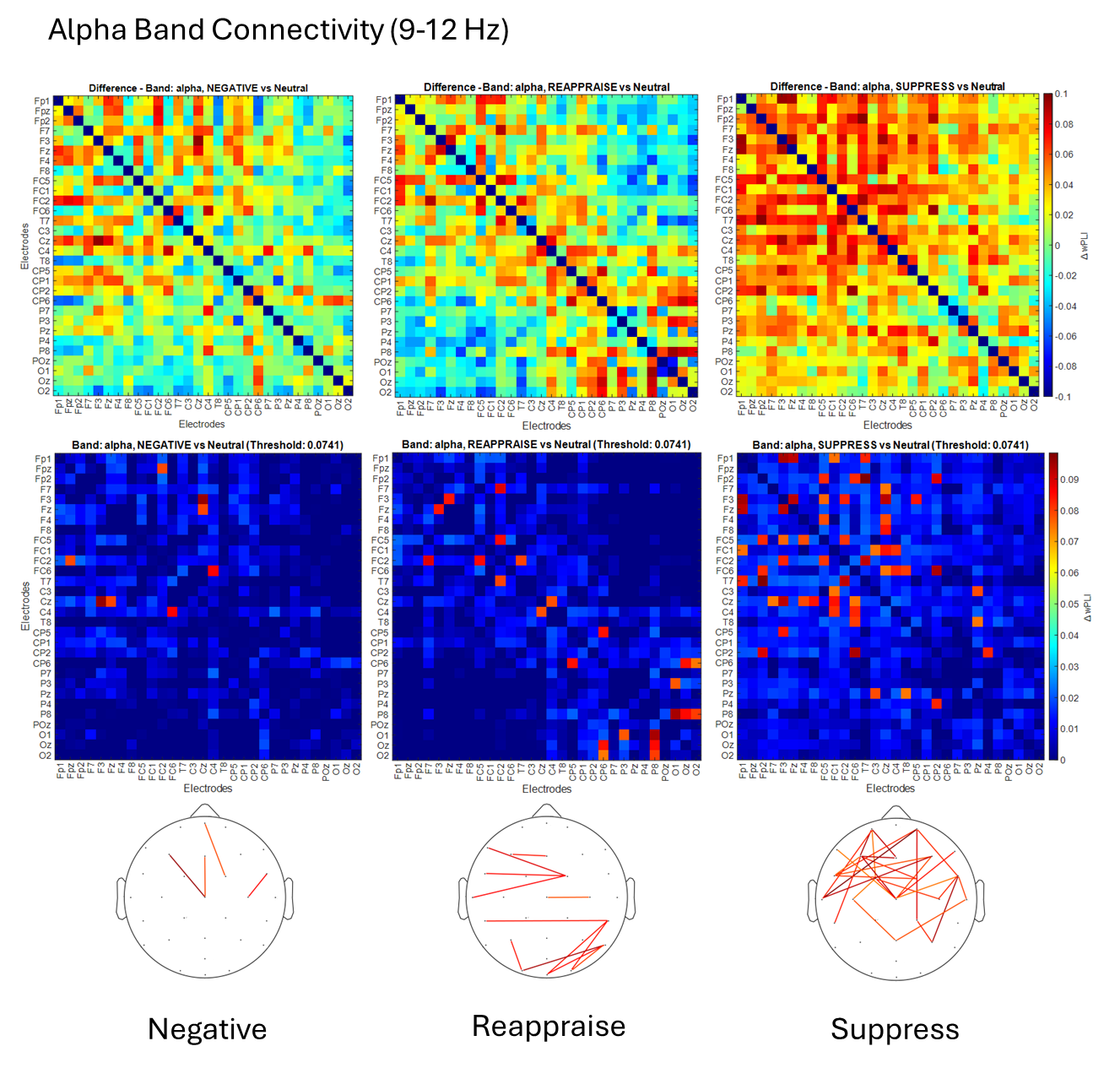


**Figure S2. Alpha Band Connectivity (9–12 Hz) During Emotion Regulation Conditions.** ***Top row***: Group-level difference matrices showing changes in debiased weighted Phase Lag Index (wPLI) connectivity in the alpha band for each emotion regulation condition (Negative, Reappraise, Suppress) relative to the Neutral baseline. Warmer colors indicate stronger increases in phase synchrony between electrode pairs (ΔwPLI). Colorbar range: –0.1 to 0.1. ***Middle row***: Thresholded difference matrices based on the 95th percentile of all positive differences across participants and conditions. Only electrode pairs with ΔwPLI exceeding the data-driven threshold (0.0741) are shown, with values below this cutoff attenuated or masked. Colorbar range: 0 to 0.09. ***Bottom row***: Scalp maps depicting the topographical distribution of suprathreshold functional connections in each condition. Colored lines represent electrode pairs showing increased alpha-band connectivity relative to Neutral, with warmer hues reflecting higher ΔwPLI values. These results reveal widespread alpha-phase synchronization during suppression, moderate connectivity increases under reappraisal, and more localized effects during passive viewing of negative stimuli.
